# Supplementary material for: Culturally adapted pulmonary rehabilitation for adults living with post-tuberculosis lung disease in Kyrgyzstan: protocol for a randomised controlled trial with blinded outcome measures
Source: BMJ Open. 2022 Feb 18;12(2):e048664. doi: 10.1136/bmjopen-2021-048664 (PMC8860058; doi:10.1136/bmjopen-2021-048664)
Supplement: Supplementary data [file bmjopen-2021-048664supp001.pdf]

APPENDIX A: Pulmonary Rehabilitation Satisfaction Survey

Please tick the relevant column for your answer to each statement below:	**Strongly Disagree**	**Disagree**	**Neutral**	**Agree**	**Strongly Agree**
I can do more of my daily activities since completing pulmonary rehabilitation.					
My levels of fitness have improved since beginning pulmonary rehabilitation.					
I have found pulmonary rehabilitation to be worthwhile.					
The information in the education talks was useful.					
Pulmonary rehabilitation has helped me to manage my lung condition more effectively.					
I would recommend this pulmonary rehabilitation course to others with a lung condition.					

- **What were the most useful aspects of the course?**

- **Is there anything you feel we could add to the course?**

- **Do you have a comment that that we could use for promotion of the programme which would encourage other patients to participate?**

Thank you very much for taking the time to complete this survey.

Supplementary Table 1. Schedule of PR sessions

Components & duration	Week 1		Week 2		Week 3	
	**Class 1**	**Class 2**	**Class 3**	**Class 4**	**Class 5**	**Class 6**
Education and tea (35 min): • Lectures (15 min) • Questions and feedback (10 min) • Tea (10 min)	Drug treatment and the use of inhalers, as well as why they are prescribed	Healthy nutrition and its role in human life	Strategies for coping with PTBLD	Chest infections and what to do if symptoms worsen	Pulmonary rehabilitation: continuation of the programme at home	Question and Answer session
Upper body resistance training	Weights, Theraband	Weights, Theraband	Weights, Theraband	Weights, Theraband	Weights, Theraband	Weights, Theraband
Lower body resistance training	Sit-to-stand, stepping up-down	Sit-to-stand, stepping up-down	Sit-to-stand, stepping up-down	Sit-to-stand, stepping up-down	Sit-to-stand, stepping up-down	Sit-to-stand, stepping up-down
Aerobic training	Walking, cycling	Walking, cycling	Walking, cycling	Walking, cycling	Walking, cycling	Walking, cycling
**Components & duration**	**Week 4**		**Week 5**		**Week 6**	
	**Class 7**	**Class 8**	**Class 9**	**Class 10**	**Class 11**	**Class 12**
Education and tea (35 min): • Lectures (15 min) • Questions and feedback (10 min) Tea (10 min)	Information about the respiratory system and possible causes of shortness of breath	What is tuberculosis and how does it affect the lungs	Your experience of living with and after TB	Co-diseases and their impact	Pulmonary rehabilitation: the role of exercise in building strength and endurance	Cigarette smoking and exposure to biomass smoke
Upper body resistance training	Weights, Theraband	Weights, Theraband	Weights, Theraband	Weights, Theraband	Weights, Theraband	Weights, Theraband
Lower body resistance training	Sit-to-stand, stepping up-down	Sit-to-stand, stepping up-down	Sit-to-stand, stepping up-down	Sit-to-stand, stepping up-down	Sit-to-stand, stepping up-down	Sit-to-stand, stepping up-down
Aerobic training	Walking, cycling	Walking, cycling	Walking, cycling	Walking, cycling	Walking, cycling	Walking, cycling

Supplementary Table 2. Physical activity data collection and accelerometry processing criteria

Criteria	Details
Accelerometer Model	ActiGraph wGT3X-BT (version 6.13.4; firmware 1.9.2)
Serial number range	Twenty unique devices will be used; ranging from MOS2E09190617 to MOS2E25190750 and averaging six deployments per device (same serial used for baseline and follow-up wear periods to remove any inter-device variability)
Piezosensor orientation	Triaxial
Mode setup	Mode 29 (x, y, z, steps, lux)
Original sample rate	100 Hz (.gt3x file format)
Deployment method	Baseline:<br>Fitted by research team on Day 0 (Baseline PR Assessment)<br>Fitted by participant on Day 1<br>Follow-up:<br>Fitted by research team Day 0 (11th/12th session)<br>Fitted by participant on Day 1
Location worn	Anterior hip adjacent to the mid-line of the thigh
Requested days of wear	7 days of free-living (10,080 epochs)
Initialization	Not deployed in delay mode in order to standardised capture of Day 0 (00:00) with stop time based on date of first PR class (baseline) and date of follow-up assessment
Wear instructions	Wear continuously except for sleep and water-based activities
Non-wear appropriation	≥60 min of consecutive 0s with allowance for 2 minutes of interruptions
Valid day criteria	≥8 hours of valid waking wear time
Valid file	≥4 valid days for each of the two time points
Missing data	Data modelling or imputation will not be performed
Epoch length	60 seconds
Intensity classification (absolute)	Uniaxial (x-axis) intensity cut-points as follows: Stationary <100 cpm; Light 100-2019 cpm; Moderate 2020-5998cpm; Vigorous ≥5999cpm (Moderate-to-vigorous ≥2020 cpm)
Intensity classification (relative)	Uniaxial (x-axis) cut-points based on Endurance Shuttle Walk Test performance
