# Supplementary material for: Culturally adapted pulmonary rehabilitation for adults living with post-tuberculosis lung disease in Kyrgyzstan: protocol for a randomised controlled trial with blinded outcome measures
Source: BMJ Open. 2022 Feb 18;12(2):e048664. doi: 10.1136/bmjopen-2021-048664 (PMC8860058; doi:10.1136/bmjopen-2021-048664)
Supplement: Author's manuscript [file bmjopen-2021-048664.draft_revisions.pdf]

BMJ Open

BMJ Open is committed to open peer review. As part of this commitment we make the peer review history of every article we publish publicly available.

When an article is published we post the peer reviewers' comments and the authors' responses online. We also post the versions of the paper that were used during peer review. These are the versions that the peer review comments apply to.

The versions of the paper that follow are the versions that were submitted during the peer review process. They are not the versions of record or the final published versions. They should not be cited or distributed as the published version of this manuscript.

BMJ Open is an open access journal and the full, final, typeset and author-corrected version of record of the manuscript is available on our site with no access controls, subscription charges or pay-per-view fees (http://bmjopen.bmj.com).

If you have any questions on BMJ Open's open peer review process please email info.bmjopen@bmj.com

BMJ Open

Culturally adapted Pulmonary Rehabilitation for adults living with post-tuberculosis lung disease in Kyrgyzstan: Protocol for a randomised controlled trial with blinded outcome measures

Journal:	*BMJ Open*
Manuscript ID	bmjopen-2021-048664
Article Type:	Protocol
Date Submitted by the Author:	04-Jan-2021
Complete List of Authors:	Akylbekov, Azamat; National Center of Cardiology and Internal Medicine named after academician M. Mirrakhimov, Pulmonology<br>Orme, Mark; University of Leicester, Respiratory Sciences<br>Jones, Amy; University of Leicester, Respiratory Sciences<br>Mademilov, Maamed; National Center of Cardiology and Internal Medicine named after academician M. Mirrakhimov, Pulmonology<br>Muratbekova, Aibermet; National Center of Cardiology and Internal Medicine named after academician M. Mirrakhimov, Pulmonology<br>Aidaralieva, Shoira; National Center of Cardiology and Internal Medicine named after academician M. Mirrakhimov, Pulmonology<br>Mirzalieva, Gulzada; National Center of Cardiology and Internal Medicine named after academician M. Mirrakhimov, Pulmonology<br>Oleinik, Alena; National Center of Cardiology and Internal Medicine named after academician M. Mirrakhimov, Pulmonology<br>Magdieva, Kamila; National Center of Cardiology and Internal Medicine named after academician M. Mirrakhimov, Pulmonology<br>Taalaibekova, Aijan; National Center of Cardiology and Internal Medicine named after academician M. Mirrakhimov, Pulmonology<br>Rysbek kyzy, Aidai; National Center of Cardiology and Internal Medicine named after academician M. Mirrakhimov, Pulmonology<br>Yusuf, Zainab; Centre for Exercise and Rehabilitation Science, NIHR Leicester Biomedical Research Centre-Respiratory, University Hospitals of Leicester NHS Trust; University of Leicester, Department of Respiratory Sciences<br>Rupert, Jones<br>Barton, Andy; South West Research Design Service,<br>Miah, Ruhme ; University of Leicester, Department of Respiratory Sciences<br>Manise, Adrian ; University of Leicester, Department of Respiratory Sciences<br>Matheson, Jesse; University of Sheffield, Department of Economics<br>Malcolm, Dominic; University of Loughborough<br>Free, Robert; Leicester NIHR Biomedical Research Centre (Respiratory theme), ; University of Leicester, Infection, Inflammation and Immunity<br>Steiner, Michael; Institute for Lung Health, NIHR Leicester Biomedical Research Centre – Respiratory, University of Leicester, Glenfield Hospital,<br>Sooronbaev, Talant; National Center of Cardiology and Internal Medicine

	named after academician M. Mirrakhimov, Pulmonology<br>Singh, Sally; University Hospitals of Leicester NHS Trust, Cardiac/Pulmonary Rehabilitation

Keywords:	Rehabilitation medicine < INTERNAL MEDICINE, RESPIRATORY MEDICINE (see Thoracic Medicine), Respiratory infections < THORACIC MEDICINE, Tuberculosis < INFECTIOUS DISEASES, Thoracic medicine < INTERNAL MEDICINE, INTERNAL MEDICINE

SCHOLARONE™ Manuscripts

Culturally adapted Pulmonary Rehabilitation for adults living with post-tuberculosis lung disease in Kyrgyzstan: Protocol for a randomised controlled trial with blinded outcome measures

Azamat Akylbekov[1], Mark W. Orme[2,3], Amy V. Jones[2,3], Maamed Mademilov[1], Aibermet Muratbekova[1], Shoira Aidaralieva[1], Gulzada Mirzalieva[1], Alena Oleinik[1], Kamila Magdieva[1], Aijan Taalaibekova[1], Aidai Rysbek kyzy[1], Zainab K. Yusuf[2,3], Rupert Jones[4], Andy Barton[4], Ruhme Miah[2,3], Adrian Manise[5], Jesse Matheson[6], Dominic Malcolm[7], Robert C. Free[2], Michael C. Steiner[2,3], Talant Sooronbaev[1] and Sally J. Singh[2,3]

Affiliations

1. National Institute for Cardiology and Internal Medicine (NCCIM), Bishkek, Kyrgyzstan

2. Department of Respiratory Sciences, University of Leicester, United Kingdom

3. Centre for Exercise and Rehabilitation Science (CERS), NIHR Leicester Biomedical Research Centre – Respiratory, University Hospitals of Leicester NHS Trust – Leicester, United Kingdom

4. Faculty of Health, University of Plymouth, United Kingdom

5. NIHR Leicester Biomedical Research Centre, University Hospitals of Leicester NHS Trust – Leicester, United Kingdom

6. Department of Economics, University of Sheffield, Sheffield, United Kingdom

7. School of Sport, Exercise and Health Sciences, Loughborough University

Protocol Version 1 23/10/2019

ABSTRACT

Introduction: Pulmonary rehabilitation (PR) is a programme of individually prescribed physical exercise, education, and self-management activities. PR is recommended in international guidelines for managing COPD and other chronic respiratory diseases. PR is still under-recognised in tuberculosis (TB) guidelines and PR is not available in many low- and middle income countries (LMIC) and for people with post-TB lung disease (PTBLD). The main aims of the study are to adapt and define a culturally appropriate PR programme in Kyrgyzstan for people living with PTBLD and to test, in a fully powered randomised controlled trial (RCT), the effectiveness of PR in improving exercise capacity for people living with PTBLD.

Methods and analysis: The study will be divided into three stages: *Stage 1:* Focus group discussions with patients living with PTBLD and interviews with PR referrers will be conducted to explore initial perceptions and inform the cultural adaptation, structure and content of PR. *Stage 2a:* A single-blind RCT evaluating the effectiveness of a culturally-adapted 6 week PR programme on maximal exercise capacity, assessed by the incremental shuttle walking test, before and after PR. Participants will be additionally followed-up 12 weeks post-baseline. Additional outcomes will include health-related quality of life, respiratory symptoms, psychological wellbeing and physical function. *Stage 2b:* Participants' experience of PR will be collected through interviews and using a log book and a patient evaluation form. Staff delivering PR will be interviewed to explore their experience of delivering the intervention and refining the delivery for future implementation.

Ethics and dissemination: The study was approved 22/07/2019 by Ethics Committee NCCIM (ref: no. 17) and by University of Leicester ethics committee (ref: no. 22293). Study results will be disseminated through appropriate peer-reviewed journals, national and international respiratory/physiotherapy conferences, social media, and through patient and public involvement events in Kyrgyzstan and in the UK.

Trial registration: International Standard Randomised Controlled Trials Numbers: ISRCTN11122503

Main author's contact email: azamatti@yahoo.com

Strengths and limitations of this study

- This project will inform an appropriate pulmonary rehabilitation programme for people living with post-tuberculosis lung disease (PTBLD) in Kyrgyzstan. This study is the first fully powered randomised controlled trial (RCT) of a culturally appropriate pulmonary rehabilitation programme for adults with PTBLD in Central Asia.
- This is a single-centre fully powered RCT; whilst findings will be important for assessing impact of pulmonary rehabilitation in Kyrgyzstan, Central Asia and low- and middle-income countries (LMIC) more broadly, findings may not be generalizable to other parts of the world.
- Taking a mixed-method approach, this study will provide a rich insight into the experiences of pulmonary rehabilitation for people living with PTBLD.
- Using a common set of outcome measures specifically designed for pulmonary rehabilitation in LMIC will allow for direct comparisons of outcomes between sites across the world.

INTRODUCTION

Background and rationale

Tuberculosis (TB) is an infectious disease and a major cause of death worldwide [1]. It typically affects the lungs (pulmonary TB) and approximately a quarter of people globally are at risk of developing TB [1]. Every year approximately 10 million people worldwide develop TB [2], of which 500,000 are multi-drug resistant cases [3]. Pulmonary TB remains an important cause of chronic respiratory impairment in low- and middle-income countries (LMIC) and globally. Kyrgyzstan is classified as lower-middle income country, is situated in Central Asia and has a higher respiratory mortality compared to Europe, with a TB incidence of 100:100,000. [4]

The presence of long-term respiratory sequelae following Pulmonary TB treatment is well established. [5] [6] The persistence of abnormal airway physiology [7], specifically fixed airways obstruction after treatment, has been documented in large population-based cross-sectional studies. The consequence of suffering from Pulmonary TB is termed post-TB lung disease (PTBLD). PTBLD negatively impacts people's quality of life, with exercise intolerance, respiratory symptoms, and abnormal lung function. [8][9] PTBLD also poses a significant economic burden to individuals and societies, not only attributable to direct costs of drugs and hospital stay, but also indirect costs such as missed workdays or lower productivity. The burden of PTBLD alongside the economic conditions in LMIC such as Kyrgyzstan aggravates the situation of patients, leading to disability and early mortality. [10]

Pulmonary Rehabilitation (PR) is a programme of individually prescribed physical exercise, education, and self-management activities. PR is recommended in international guidelines for managing COPD and other lung diseases [11] but is still under-recognised in TB guidelines and in LMIC. [12] With the established benefits of PR for people living with chronic respiratory diseases many high-income countries have integrated PR services within routine healthcare services. Delivery of PR is encouraged in LMIC where there is profound need [13], where there is emerging evidence that it is feasible to deliver PR in low-resource settings [14] [15], including for people living with PTBLD [23] for whom post-treatment support for health and wellbeing is needed. [16] Indeed, PR is typically delivered by a multidisciplinary team which has been advocated for people living with PTBLD in order to account for psychosocial and economical challenges. [17] [18] Western models of PR may not be optimal in LMIC and there is a need to implement PR interventions tailored to the local culture, traditions, geography, population, and healthcare systems. To date, despite the potential to improve patient outcomes in PTBLD there has been no formal fully-powered trial of PR for people living with PTBLD in LMIC. It is important for PR to be appealing to patients and to ensure it is delivered in a manner sensitive to local contexts. Accordingly, the aim of this study is to adapt a conventional model of PR to Kyrgyzstan and PTBLD contexts and test its effectiveness in a fully powered randomised controlled trial (RCT).

For peer review only

The trial is comprised of: Stage 1: Adaptations to PR informed by patients living with PTBLD and healthcare professionals (HCPs) who would refer to PR; Stage 2a: fully powered RCT; and Stage 2b: qualitative evaluation of PR. The objectives of this study are to:

1. Explore the views of people living with PTBLD and HCPs (referrers and deliverers) involved in the care of patients living with PTBLD to inform the adaptations needed for a PR programme suitable for people living with PTBLD in Kyrgyzstan.
2. Conduct a single-blind fully powered RCT to assess the effectiveness of adapted 6-week PR on maximal exercise capacity (assessed by the incremental shuttle walking test (ISWT)).
3. Evaluate a range of secondary outcome measures including health-related quality of life, respiratory symptoms, functional status and psychological wellbeing following PR.
4. Assess any further changes in all outcome measurements 12 weeks post-baseline.
5. Assess the acceptability of PR of participants and the staff delivering PR to inform further improvements to the service.

METHODS AND ANALYSIS

Trial design

The trial will be conducted, analysed and reported according to the Standard Protocol Items: Recommendations for Interventional Trials (SPIRIT) statement and the trial has been prospectively registered (ISRCTN11122503).

Stage 1

Focus groups with adults living with PTBLD and interviews with staff who would be involved in referring patients to PR will be conducted to explore initial perceptions of PR and to inform the cultural adaptation of PR, for their insight, any minor modifications and any additional needs so that it is suitable for this group and for understand their views on any specific topics for the health education component, or any types of exercises that are suitable for enjoyment and adherence for this population.

Stage 2a

After the PR programme has been adapted based on the qualitative work at stage 1, a RCT evaluating the effectiveness of PR in adults with PTBLD. Adults living with PTBLD will be randomly allocated into one of two groups: Group A (assigned PR programme) or Group B (usual care on PR waiting list).

Stage 2b

Upon completion of PR, patient focus groups and interviews with staff who were involved in the delivery of PR will be conducted. Participants in the PR group will be asked to log their experience as they progress through the programme by completing a log book accessible before, during and after sessions, as well as a dedicated patient evaluation form to be completed after their last class.

Study setting

The study will be conducted at the National Center for Cardiology and Internal Medicine (NCCIM), Bishkek, Kyrgyzstan. Interviews will be conducted in quiet and familiar settings, for example a HCP's office. Spacious and quiet rooms will be used for focus groups. For the PR program, a specially allocated room with the necessary equipment and air conditioner will be used. For all stages of the project, appropriate precautions will be taken in line with local COVID-19 guidance.

Participants

For Stage 1, eligible patients will be: aged ≥18 years; confirmed diagnosis of a TB-negative patients with PTBLD using a Ziehl-Nielsen stain or GeneExpert method and completed TB treatment. Eligible HCPs will be directly involved in the long-term care of PTBLD patients.

For peer review only

For Stage 2a, the inclusion criteria for patients will be: aged ≥18 years; confirmed diagnosis of a TB-negative patients with PTBLD using a Ziehl-Nielsen stain or GeneExpert method; completed TB treatment; Medical Research Council (MRC) dyspnoea score grade 2 or higher. Exclusion criteria for patients will be: Comorbidities such as severe or unstable cardiovascular, other internal diseases and locomotor difficulties that preclude exercise; malignant disease such as lung cancer; evidence of active TB on Chest X-ray or sputum tests within 1 month of assessment; unable or unwilling to provide informed consent.

For Stage 2b, all patients consented and randomised to receive PR and all staff involved in the delivery of PR to patients as part of the trial will be eligible.

Procedure

After receiving a research fact sheet, patients eligible for the study will be asked if they would like to participate after they have had the opportunity to ask questions. If they wish to take part, they will be asked to provide written informed consent (Appendix A). Reasons for declining the study will be taken as field notes. If they wish to participate, they will be asked to provide written informed consent. Reasons for declining to participate will be reflected in the field notes. The staff of the family medicine centres, doctors of the City Tuberculosis Hospital, the City Tuberculosis Dispensary, the National Hospital, District Hospitals and the National Centre of Cardiology and Internal Medicine will screen patients for eligibility and refer interested patients to the PR study team. Interested patients will be screened by the study team to confirm interest in participation and to schedule the baseline visit. After providing written informed consent, participants will undergo a baseline assessment at the NCCIM by a specially trained team of researchers and residents. After the baseline measures are complete, participants will be randomised into one of two groups: PR or usual care control. At 6-weeks post-baseline and 12-weeks post-baseline, patients will undergo follow-up assessments. If participants are not able to attend follow-up assessments in person, questionnaires only will be completed via telephone appointment. Experiences of the patients living with PTBLD and PR deliverers will be explored in interviews and focus groups. Participants who drop out of the trial will be asked to take part in an interview and information provided freely by the participants will be collected as field notes. All outcome measures will be performed at 6 and 12 weeks by dedicated staff who are blinded to group allocations. Control participants will be offered PR at the end of the study (see Figure 1).

Intervention

The PTBLD PR programme will be held twice-weekly for a period of 6-weeks. Each session will last approximately two hours, consisting of exercise and health education (Supplementary Table 1). The PR programme recruitment will be rolling rather than fixed groups, with a maximum capacity of five patients per session (due to pandemic), and supervised by a nurse, doctor, researcher or physiotherapist. The equipment used will be simple and based on local availability. It will be based on the core elements of an evidence-based rehabilitation, including exercises and health education but we will be adapting the detail and delivery based on the qualitative work in Stage 1.

Core components of PR exercise are upper and lower body resistance training and aerobic training will be informed by international guidelines. [11] [19] The strength exercises will include (at least) bicep curls, pull-ups, sit-to-stands and step ups. Patients will be asked to do one session of strength training and to walk every day at home in addition to structured classes. Minimal equipment will be used where possible, such as bottles filled with water, to achieve the desired weight in kilograms and to offer insight into how participants can exercise effectively at home during and after finishing PR. Aerobic exercise will include walking and static cycling. Walking will be individually prescribed at a speed equivalent to 85% maximal oxygen capacity (VO_2) peak derived from the ISWT. [20] [21] Walking will be monitored and target duration increased by the instructor as the programme progresses. The content delivered within the education sessions will be based on existing materials, with refinement from Stage 1 of this trial and will be delivered by an interdisciplinary team.

Usual care

For peer review only - http://bmjopen.bmj.com/site/about/guidelines.xhtml

As part of usual care, participants will receive their usual prescription medications (if appropriate), and an educational booklet regarding PTBLD and the importance of exercise, healthy diet, smoking cessation and avoiding biomass smoke.

Sample size

Stage 1

We will conduct 8-10 focus groups of 2-10 patients (ideally 6-8 patients) in each group and up to 15 interviews with referrers to explore initial perceptions of PR and to inform the cultural adaptation of PR, for their insight, any minor modifications and any additional needs so that it is suitable for this group and for understand their views on any specific topics for the health education component, or any types of exercises that are suitable for enjoyment and adherence for this population.

Stage 2a

Assuming a statistical significance level of 5% and a statistical power of 80%, in order to detect a 35m difference in ISWT measured at baseline and after completion of PR, [22] a total of 114 participants are required (PR: 57 participants or waiting list control: 57 participants). This power calculation was performed using a paired t-test and assumes the ISWT follows an approximately normal distribution. Data from a feasibility study in Uganda, assessing PR in a PTBLD population has guided this calculation. [14] Conservatively assuming up to 30% loss to follow-up at 6-weeks, this will require up to 163 participants to be recruited and randomised (1:1) to each arm. The estimated 70% ineligibility rate suggests approximately 543 PTBLD patients will be screened for this trial.

Stage 2b

We will conduct up to 5 focus groups of 2-10 patients (ideally 6-8 patients) and an interview with all members of the PR delivery team. All participants randomised to the PR group will be asked to complete the log book and evaluation form.

Randomisation

Randomisation will occur through a web based service Sealed Envelope™ and conducted in a designated room by a dedicated member of the research team. Participants will be randomised (1:1) to either PR or usual care. A dedicated member of the research team will inform the assessors of group allocations via telephone. Participants will be informed at the end of their baseline visit to arrange their first PR class.

Blinding

Outcome measures taken at baseline, post-PR, and follow-up will be taken by a researcher blinded to group allocation. It is not possible to blind participants to treatment due to the nature of a PR intervention. Research staff will be blinded to outcome measures. Participants will be advised not to reveal their group during the follow-up assessments. Episodes of un-blinding will be documented and reported for both time points.

Qualitative data collection

All interview and focus group discussions will be audio-recorded, expected to last on average 20-40 minutes, and will be conducted by a trained interviewer. Recordings will be transcribed verbatim, with identifiable information removed. Consent will be obtained from participants prior to their involvement. PR deliverers will be invited to participate in in-depth interviews at the end of the study to discuss aspects of PR, such as insights into barriers to attendance, logistical barriers of running a PR programme and their views of patients' experiences of the intervention.

Book of testimonies and evaluation form

For peer review only

Participants within PR will be asked to log their experience of PR as they progress through the programme. This will be in the form of a PR log book accessible to participants before, during and after sessions, as well as a dedicated evaluation form (Appendix B). Staff involved in PR will also receive the same evaluation form at the end of the study.

Quantitative data collection

Baseline characteristics will comprise patient socio-demographics (age, sex), age of leaving full time education, anthropometrics (height, weight, body mass index), smoking status and pack years, biomass fuel exposure, primary respiratory diagnosis, secondary respiratory diagnosis (if appropriate), spirometry data (post bronchodilation Forced Expiratory Volume in 1-second [FEV_1], post bronchodilation Forced Vital Capacity [FVC] and FEV_1/FVC ratio) (EasyOne™ Plus spirometer, NDD Medical Technologies, Switzerland); number of hospitalisations in the last 12 months, comorbidities, treatment and previous participation in PR. The height will be measured by stadiometer and the weight will be measured by electronic scales.

Outcomes

A schedule of events is provided in Table 1 and measures are in accordance with the recommended minimum dataset for PR in LMIC. [23] In the case a face-to-face follow up appointment at 12 weeks post-baseline cannot be conducted, questionnaires will be collected over telephone.

Primary outcome

The primary outcome of exercise capacity will be measured using the ISWT. [20] The ISWT will require patients to walk up and down a 10-meter course marked out using cones at walking speeds dictated by an audio signal played on an audio device. Each participant will receive standardised instructions to: "Walk at a steady pace, aiming to turn around when you hear the signal. You should continue to walk until you feel that you are unable to maintain the required speed without becoming unduly breathless". To account for any learning effect, a practice ISWT will be performed and the greatest distance walked from either test will be taken forward. The test will be terminated when either: (i) the patient indicates that they are unable to continue, (ii) the operator determines that the patient is not fit to continue or (iii) the operator assesses that the patient was unable to sustain the speed and cover the distance to the cone prior to the beep sounding.

Secondary outcomes

Exercise capacity and muscular strength

Participants will be asked to complete a five repetition sit to stand test, [24] as an indication of lower extremity strength. The time taken for participants to complete this task will be recorded.

The endurance shuttle walk test (ESWT) is a constant load walking test, in which a participant is required to walk at 85% of their maximal ISWT walking speed. An audio signal is played to the participant and they are required to walk around a 10-meter course marked by cones. [21]

Symptoms

The MRC Dyspnoea Scale will be used to assess dyspnoea. [25] This is a 5-point questionnaire, in which the participant self-reports their dyspnoea. Chest pain will be assessed using four questions based on Brief Pain Inventory (Short Form). [26]

Quality of life

The Clinical COPD Questionnaire (CCQ) [27] is a 10-item health related quality of life questionnaire that is divided into three domains: symptoms, functional and mental. The COPD Assessment Test (CAT) [28] is a self-administered questionnaire that is used to quantify the symptom burden of COPD. The questionnaire consists

For peer review only

of eight items, each with a 6-point scale, creating a total score out of 40. The EQ5D5L [29] will be used to assess generic health-related quality of life, and comprises of five dimensions: mobility, self-care, usual activities, pain/discomfort and anxiety/depression. The questionnaire also contains a visual analogue scale, where the participant identifies their health from 'the best you can imagine' and the 'worst you can imagine'.

Psychological wellbeing

The self-reported Hospital Anxiety and Depression Scale [30] will be used to determine levels of anxiety and depression. The questionnaire consists of 14 questions with a 4-point Likert scale, and two 7-item subscales for anxiety and depression.

Productivity

The Work Productivity and Activity Impairment (WPAI) questionnaire [31] will be used to assess productivity and impairments in paid/ unpaid work and activities. The questionnaire is self-reported and participants are asked to recall the time missed from work in the last seven days.

Physical activity

Physical activity will be assessed using an ActiGraph wGT3X-BT activity monitor (ActiGraph, Pensacola, FL, USA). Participants will be asked to place the device on their right anterior hip during waking hours for one week prior to attending PR (baseline) and for one week prior to their 6-week follow-up visit. Participants will be asked to remove the device for water-based activities and for sleep. Written instructions will be provided to the participants. Full methodology for the assessment of physical activity is provided in Supplementary Table 2.

Cost/benefit analysis

The cost of the PR programme will be calculated, including single and recurrent costs. Single costs will include the necessary costs to set up and run PR and the recurrent costs will include any item with a life expectancy of no more than 1 year e.g. disposable materials. Fixed costs will be captured before the first patient being recruited into the trial. Recurrent costs will be collected at the halfway stage of recruitment. An average cost of PR per patient will be calculated.

Data management

Data collected during the study will be entered onto a database using Research Electronic Data Capture (REDCap), [32] [33] hosted by University of Leicester, UK. Access to the database will be via a secure password protected web-interface. Data will be validated using real-time data entry validation and electronic checks, led by the Independent Data Monitoring Committee (IDMC) at the University of Leicester, UK. The participants will be allocated a unique study-specific identification code.

Data analysis

For Stages 1 and 2b, qualitative data will be transcribed verbatim in Russian and translated into English for triangulation with UK-based researchers. Transcripts will be analysed using Thematic Analysis; following the six distinct stages of familiarization with data; generating initial codes; searching for themes; reviewing themes; defining and naming themes and producing the report. [34] [35] The qualitative lead will carry out initial coding and a sample of the transcripts will be coded independently by a second researcher to improve consistency and interpretive authenticity. The team will meet regularly to review emerging themes, with close attention to interactions within the interview and focus group data.

For Stage 2a, quantitative analysis will be conducted using Statistical Package for the Social Sciences. Data will be reported as mean (standard deviation (SD)), median (interquartile range (IQR)) or frequency (%) as

For peer review only

appropriate. All randomised patients will be included in an intention to treat analysis with the primary efficacy analysis based on both per protocol and modified intention-to-treat populations, with missing data imputed. For the primary analysis, the differences in the primary outcome of walking distance on the ISWT will be estimated using mixed models (unadjusted and adjusted for covariates if there are differences between groups at baseline). There will be no formal interim analysis of the data.

Adverse events

All adverse events and serious adverse events will be recorded on an adverse event log. This will be recorded within the study trial management paperwork, CRF and study database. The IDMC will review high level safety data which will be monitored at least every month and on an ad hoc basis as required. The NIHR RECHARGE Scientific Committee will be informed of all adverse events and determine the need to terminate the trial prematurely. Participants who experience any such event will receive the appropriate care.

Patient and public involvement

We know from conversations with adults living with PTBLD that support after completing TB treatment is lacking and that these individuals continue to live with a reduce quality of life, including respiratory symptoms and social isolation. It is clear that these individuals would benefit from support to better manage their condition, which is what this project seeks to deliver. The PR intervention and trial are to be informed directly from people living with PTBLD and healthcare workers in Kyrgyzstan. The results of this work will be presented to patients and the public at dedicated dissemination events, including those hosted at the NCCIM, Bishkek and surrounding TB Hospitals.

ETHICS AND DISSEMINATION

Research ethics approval

The study was approved 22/07/2019 by Ethics Committee of the NCCIM (ref: no. 17) 3, Togolok Moldo Street, Bishkek, Kyrgyzstan. Ethics approval was also provided by the University of Leicester ethics committee on the 16/09/2019 ((reference number 22293, ethicsapp@leicester.ac.uk). Information about the study participants will be strictly confidential, and the names will be used only for internal reporting. No identifiable data will be published in any article.

Dissemination policy

Study results will be disseminated through appropriate peer-reviewed journals, national and international conferences, and through social media. All participants will be provided a summary of the trial results. In February 2021, a National Scientific Medical Forum is planned and a National Respiratory Congress is planned in October 2021, which will also present the results of the study and disseminate information about the project. In addition, mobile rehabilitation schools are planned during 2021.

AUTHORS' CONTRIBUTIONS

All authors (Azamat Akylbekov, Mark W. Orme, Amy V. Jones, Maamed Mademilov, Aibermet Muratbekova, Shoira Aidaralieva, Gulzada Mirzalieva, Alena Oleinik, Kamila Magdieva, Aijan Taalaibekova, Aidai Rysbek kyzy, Zainab K. Yusuf, Rupert Jones, Andy Barton, Ruhme Miah, Adrian Manise, Jesse Matheson, Robert C. Free, Michael C. Steiner, Talant Sooronbaev, Sally J. Singh) have substantially contributed to the conception and design of the study. Azamat Akylbekov, Mark W. Orme and Amy V. Jones drafted the manuscript. All authors of the paper have revised the content and approved the final version to be published. All authors are accountable for all aspects of the work.

ROLES AND RESPONSIBILITIES OF AUTHORS

a) Substantially contributed to the conception and/ or design of the work

For peer review only - http://bmjopen.bmj.com/site/about/guidelines.xhtml

b) Substantially contributed to the acquisition, analysis, or interpretation of data for the work

c) Substantially contributed to the drafting of the work and/ or revising it critically for important intellectual content

FUNDING STATEMENT

This research was funded by the National Institute for Health Research (NIHR) (17/63/20) using UK aid from the UK Government to support global health research. The views expressed in this publication are those of the author(s) and not necessarily those of the NIHR or the UK Department of Health and Social Care.

COMPETING INTERESTS

None declared

DATA ACCESS

Following the completion of the project, data from the Global Health Research Group on Respiratory Rehabilitation (Global RECHARGE) Core Dataset will be made available to the wider community upon reasonable request.

REFERENCES

[1] "World Health Organisation. Global tuberculosis report. 2020.," 2020. https://www.who.int/tb/publications/global_report/en/.

[2] United Nations General Assembly. Resolution 73/3, "Political declaration of the high-level meeting of the General Assembly on the fight against tuberculosis. United Nations," *Int. Organ.*, vol. 9, no. 1, pp. 57–156, 2018, doi: 10.1017/s002081830002244x.

[3] M. Uplekar *et al.*, "Viewpoint WHO ' s new End TB Strategy," pp. 1799–1801, 2015, doi: 10.1016/S0140-6736(15)60570-0.

[4] WHO, "Tuberculosis profile: Kyrgyzstan," 2019. https://worldhealthorg.shinyapps.io/tb_profiles/?_inputs_&entity_type=%22country%22&lan=%22EN%22&iso2=%22KG%22.

[5] A. L. Byrne, B. J. Marais, C. D. Mitnick, L. Lecca, and G. B. Marks, "Tuberculosis and chronic respiratory disease: A systematic review," *Int. J. Infect. Dis.*, vol. 32, pp. 138–146, 2015, doi: 10.1016/j.ijid.2014.12.016.

[6] B. W. Allwood, L. Myer, and E. D. Bateman, "A systematic review of the association between pulmonary tuberculosis and the development of chronic airflow obstruction in adults," *Respiration*, vol. 86, no. 1, pp. 76–85, 2013, doi: 10.1159/000350917.

[7] K. Dheda, H. Booth, J. F. Huggett, M. A. Johnson, A. Zumla, and G. A. W. Rook, "Lung remodeling in pulmonary tuberculosis," *J. Infect. Dis.*, vol. 192, no. 7, pp. 1201–1210, 2005, doi: 10.1086/444545.

[8] S. Daniels, Kurt J.; Irusen, Elvis; Pharaoh, Hamilton; Hanekom, "Post-tuberculosis health-related quality of life, lung function and exercise capacity in a cured pulmonary tuberculosis population in the Breede Valley District, South Africa," *South African J. Physiother.*, no. English 2009, pp. 1–8, 2013.

[9] J. Meghji *et al.*, "Patient outcomes associated with post-tuberculosis lung damage in Malawi: A prospective cohort study," *Thorax*, vol. 75, no. 3, pp. 269–278, 2020, doi: 10.1136/thoraxjnl-2019-213808.

[10] K. Romanowski, B. Baumann, C. A. Basham, F. Ahmad Khan, G. J. Fox, and J. C. Johnston, "Long-term all-cause mortality in people treated for tuberculosis: a systematic review and meta-analysis," *Lancet Infect. Dis.*, vol. 19, no. 10, pp. 1129–1137, 2019, doi: 10.1016/S1473-3099(19)30309-3.

[11] M. A. Spruit *et al.*, "An official American thoracic society/European respiratory society statement: Key

For peer review only

concepts and advances in pulmonary rehabilitation," *Am. J. Respir. Crit. Care Med.*, vol. 188, no. 8, 2013, doi: 10.1164/rccm.201309-1634ST.

[12] A. Wanner *et al.*, "International research and guidelines on post-tuberculosis chronic lung disorders: A systematic scoping review," *BMJ Glob. Heal.*, vol. 3, no. 4, pp. 1–8, 2018, doi: 10.1136/bmjgh-2018-000745.

[13] S. J. Singh, D. M. G. Halpin, S. Salvi, B. J. Kirenga, and K. Mortimer, "Exercise and pulmonary rehabilitation for people with chronic lung disease in LMICs: challenges and opportunities.," *Lancet. Respir. Med.*, vol. 7, no. 12, pp. 1002–1004, Dec. 2019, doi: 10.1016/S2213-2600(19)30364-9.

[14] R. Jones *et al.*, "A pre – post intervention study of pulmonary rehabilitation for adults with post-tuberculosis lung disease in Uganda," pp. 3533–3539, 2017.

[15] M. Anastasaki *et al.*, "Establishing a pulmonary rehabilitation programme in primary care in Greece: A FRESH AIR implementation study," *Chron. Respir. Dis.*, vol. 16, 2019, doi: 10.1177/1479973119882939.

[16] B. Allwood *et al.*, "The long shadow post-tuberculosis," *Lancet Infect. Dis.*, vol. 19, no. 11, pp. 1170–1171, 2019, doi: https://doi.org/10.1016/S1473-3099(19)30564-X.

[17] S. Datta and C. A. Evans, "Healthy survival after tuberculosis," *Lancet Infect. Dis.*, vol. 19, no. 10, pp. 1045–1047, 2019, doi: https://doi.org/10.1016/S1473-3099(19)30387-1.

[18] T. Wingfield, M. A. Tovar, S. Datta, M. J. Saunders, and C. A. Evans, "Addressing social determinants to end tuberculosis," *Lancet*, vol. 391, no. 10126, pp. 1129–1132, 2018, doi: 10.1016/S0140-6736(18)30484-7.

[19] C. E. Bolton *et al.*, "British Thoracic Society guideline on pulmonary rehabilitation in adults.," *Thorax*, vol. 68 Suppl 2, pp. ii1-30, Sep. 2013, doi: 10.1136/thoraxjnl-2013-203808.

[20] S. J. Singh, M. D. Morgan, S. Scott, D. Walters, and A. E. Hardman, "Development of a shuttle walking test of disability in patients with chronic airways obstruction.," *Thorax*, vol. 47, no. 12, pp. 1019–1024, Dec. 1992, doi: 10.1136/thx.47.12.1019.

[21] S. M. Revill, M. D. Morgan, S. J. Singh, J. Williams, and A. E. Hardman, "The endurance shuttle walk: a new field test for the assessment of endurance capacity in chronic obstructive pulmonary disease.," *Thorax*, vol. 54, no. 3, pp. 213–222, Mar. 1999, doi: 10.1136/thx.54.3.213.

[22] R. A. Evans and S. J. Singh, "Minimum important difference of the incremental shuttle walk test distance in patients with COPD.," *Thorax*, vol. 74, no. 10, pp. 994–995, Oct. 2019, doi: 10.1136/thoraxjnl-2018-212725.

[23] M. W. Orme *et al.*, "Global RECHARGE: Establishing a standard international data set for pulmonary rehabilitation in low- and middle-income countries.," *J. Glob. Health*, vol. 10, no. 2, p. 20316, Dec. 2020, doi: 10.7189/jogh.10.020316.

[24] R. W. Bohannon, "Sit-to-stand test for measuring performance of lower extremity muscles.," *Percept. Mot. Skills*, vol. 80, no. 1, pp. 163–166, Feb. 1995, doi: 10.2466/pms.1995.80.1.163.

[25] J. C. Bestall, E. A. Paul, R. Garrod, R. Garnham, P. W. Jones, and J. A. Wedzicha, "Usefulness of the Medical Research Council (MRC) dyspnoea scale as a measure of disability in patients with chronic obstructive pulmonary disease," *Thorax*, vol. 54, no. 7, pp. 581–586, 1999, doi: 10.1136/thx.54.7.581.

[26] T. Mendoza, T. Mayne, D. Rublee, and C. Cleeland, "Reliability and validity of a modified Brief Pain Inventory short form in patients with osteoarthritis.," *Eur. J. Pain*, vol. 10, no. 4, pp. 353–361, May 2006, doi: 10.1016/j.ejpain.2005.06.002.

[27] T. van der Molen, B. W. M. Willemse, S. Schokker, N. H. T. ten Hacken, D. S. Postma, and E. F. Juniper, "Development, validity and responsiveness of the Clinical COPD Questionnaire.," *Health Qual. Life Outcomes*, vol. 1, p. 13, Apr. 2003, doi: 10.1186/1477-7525-1-13.

For peer review only

[28] P. W. Jones, G. Harding, P. Berry, I. Wiklund, W.-H. Chen, and N. Kline Leidy, “Development and first validation of the COPD Assessment Test,” *Eur. Respir. J.*, vol. 34, no. 3, pp. 648–654, 2009, doi: 10.1183/09031936.00102509.

[29] T. E. Group, “EuroQol--a new facility for the measurement of health-related quality of life.,” *Health Policy*, vol. 16, no. 3, pp. 199–208, Dec. 1990, doi: 10.1016/0168-8510(90)90421-9.

[30] A. S. Zigmond and R. P. Snaith, “The hospital anxiety and depression scale.,” *Acta Psychiatr. Scand.*, vol. 67, no. 6, pp. 361–370, Jun. 1983, doi: 10.1111/j.1600-0447.1983.tb09716.x.

[31] M. C. Reilly, A. S. Zbrozek, and E. M. Dukes, “The validity and reproducibility of a work productivity and activity impairment instrument.,” *Pharmacoeconomics*, vol. 4, no. 5, pp. 353–365, Nov. 1993, doi: 10.2165/00019053-199304050-00006.

[32] P. A. Harris, R. Taylor, R. Thielke, J. Payne, N. Gonzalez, and J. G. Conde, “Research electronic data capture (REDCap)--a metadata-driven methodology and workflow process for providing translational research informatics support.,” *J. Biomed. Inform.*, vol. 42, no. 2, pp. 377–381, Apr. 2009, doi: 10.1016/j.jbi.2008.08.010.

[33] P. A. Harris *et al.*, “The REDCap consortium: Building an international community of software platform partners.,” *J. Biomed. Inform.*, vol. 95, p. 103208, Jul. 2019, doi: 10.1016/j.jbi.2019.103208.

[34] V. Braun and V. Clarke, “Using thematic analysis in psychology,” *Qual. Res. Psychol.*, vol. 3, no. 2, pp. 77–101, 2006, doi: 10.1191/1478088706qp063oa.

[35] D. B. Maguire M, “Doing a Thematic Analysis : A Practical, Step-by-Step Guide for Learning and Teaching Scholars,” *AISHE-J.*, 2017.

For peer review only

Tables

Table 1. Schedule of events

	Baseline	6 weeks	12 weeks	
	**Face-to-face**	**Face-to-face**	**Face-to-face**	**Telephone***
Socio-demographics	x			
Age of leaving full time education	x			
Smoking and biomass exposure	x			
Respiratory diagnoses	x			
Spirometry	x			
Hospitalisations in the last 12 months	x			
Comorbidities	x			
Treatments	x			
Physical activity monitoring	x	x		
Anthropometrics	x	x	x	
ISWT and ESWT	x	x	x	
5x sit to stand	x	x	x	
MRC score	x	x	x	x
EQ5D score	x	x	x	x
Pain scores for chest pain	x	x	x	x
CCQ	x	x	x	x
CAT	x	x	x	x
HADS	x	x	x	x
WPAI questionnaire	x	x	x	x

Abbreviations: CAT – The COPD Assessment Test, CCQ – Clinical COPD Questionnaire score, ESWT – Endurance Shuttle Walk Test, HADS – Hospital Anxiety and Depression Scale, ISWT – Incremental Shuttle Walk Test, MRC – Medical Research Council, WPAI – Work Productivity and Activity Impairment Questionnaire

Figure 1 legend

Research flow. The stages and flow of research are clearly described here.

For peer review only

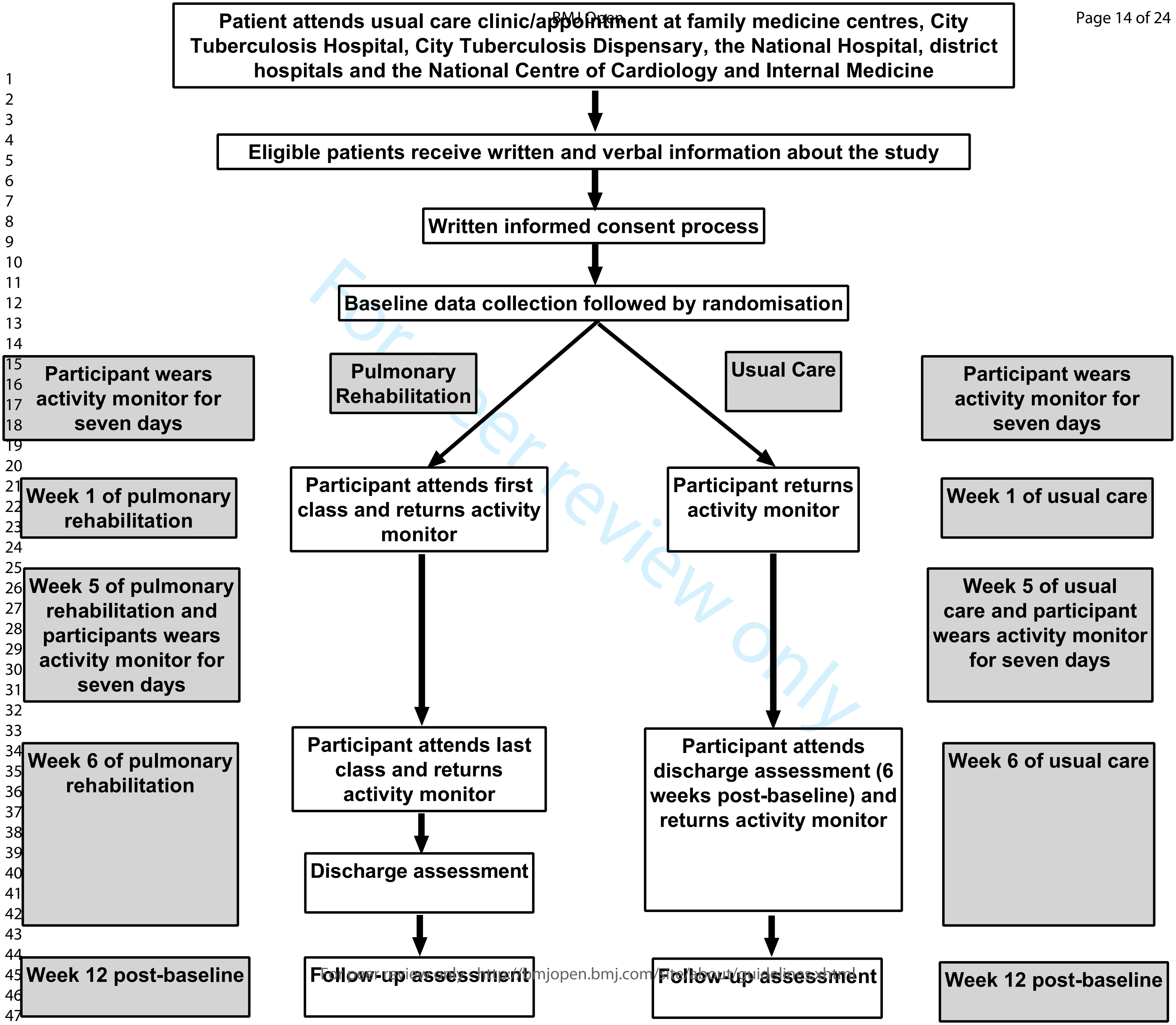

For peer review only

Appendix A: Consent form

Study number:

Title of project: Does Pulmonary Rehabilitation improve the health of people in Kyrgyzstan living with lung disease caused by tuberculosis?

Name of researcher: Prof. Talant Sooronbaev

INFORMED CONSENT FORM (Healthcare professional interview- Post) Patient identification number:

Taking Part Please initial to confirm agreement

The purpose and details of this study have been explained to me. I understand that this study is designed to further scientific knowledge and that all procedures have been approved by the University of Leicester ethics committee and the National Centre of Cardiology and Internal Medicine, Bishkek, Kyrgyzstan ethics committee.

I have read and understood the information sheet (Healthcare professional interview- Post_Version 1.0) and this consent form.

I have had an opportunity to ask questions about my participation.

I understand that taking part in the project will involve participating in an interview that will be recorded (audio or video).

I understand that personal information collected will include name and job title. I understand I will be assigned a unique study identification number and my unique identification number and personal details will be stored on a secure enrolment log.

I understand that the audio recordings taken during this interview will be converted to text but will remain anonymous.

--------------------- ---------------------

I understand that I am under no obligation to take part in the study, have the right to withdraw from this study at any stage for any reason, and will not be required to explain my reasons for withdrawing.

Use of Information

I understand that responsible persons and employees of the ethics committee will have access to my personal data for strict control and to ensure the correct conduct of the study whilst ensuring strict confidentiality will be maintained.

For peer review only

I understand this research is in collaboration with the University of Leicester, UK and all anonymised data will be shared.

I understand that all the personal information I provide will be processed in accordance with data protection legislation on the public task basis and will be treated in strict confidence unless (under the statutory obligations of the agencies which the researchers are working with), it is judged that confidentiality will have to be breached for the safety of the participant or others or for audit by regulatory authorities.

I understand that information I provide will be used in publications, reports, web pages and other academic and research outputs.

I understand that personal information collected about me that can identify me, such as my name will not be shared beyond the study team.

I agree that information I provide can be quoted anonymously in research outputs.

I give permission for the anonymised data I provide to be deposited in the data archive

governed by the National Center of Cardiology and Internal Medicine, Bishkek, Kyrgyzstan so that it can be made publicly available for future research at the end of the project.

I understand that anonymised data collected as part of this study may be used in future research.

Consent to Participate

I voluntarily agree to take part in this study. ----------------------

Name of participant	[printed]	Signature	Date
Researcher	[printed]	Signature	Date

For peer review only

APPENDIX B: Pulmonary Rehabilitation Satisfaction Survey

Please tick the relevant column for your answer to each statement below:	**Strongly Disagree**	**Disagree**	**Neutral**	**Agree**	**Strongly Agree**
I can do more of my daily activities since completing pulmonary rehabilitation.					
My levels of fitness have improved since beginning pulmonary rehabilitation.					
I have found pulmonary rehabilitation to be worthwhile.					
The information in the education talks was useful.					
Pulmonary rehabilitation has helped me to manage my lung condition more effectively.					
I would recommend this pulmonary rehabilitation course to others with a lung condition.					

- **What were the most useful aspects of the course?**

- **Is there anything you feel we could add to the course?**

- **Do you have a comment that that we could use for promotion of the programme which would encourage other patients to participate?**

Thank you very much for taking the time to complete this survey.

For peer review only

Supplementary Table 1. Schedule of PR sessions

Components & duration	Week 1		Week 2		Week 3	
	Class 1	Class 2	Class 3	Class 4	Class 5	Class 6
Education and tea (35 min):<br>• Lectures (15 min)<br>• Questions and feedback (10 min)<br>• Tea (10 min)	Drug treatment and the use of inhalers, as well as why they are prescribed	Healthy nutrition and its role in human life	Strategies for coping with PTBLD	Chest infections and what to do if symptoms worsen	Pulmonary rehabilitation: continuation of the programme at home	Question and Answer session
Upper body resistance training	Weights, Theraband	Weights, Theraband	Weights, Theraband	Weights, Theraband	Weights, Theraband	Weights, Theraband
Lower body resistance training	Sit-to-stand, stepping up-down	Sit-to-stand, stepping up-down	Sit-to-stand, stepping up-down	Sit-to-stand, stepping up-down	Sit-to-stand, stepping up-down	Sit-to-stand, stepping up-down
Aerobic training	Walking, cycling	Walking, cycling	Walking, cycling	Walking, cycling	Walking, cycling	Walking, cycling
**Components & duration**	**Week 4**		**Week 5**		**Week 6**	
	**Class 7**	**Class 8**	**Class 9**	**Class 10**	**Class 11**	**Class 12**
Education and tea (35 min):<br>• Lectures (15 min)<br>• Questions and feedback (10 min)<br>Tea (10 min)	Information about the respiratory system and possible causes of shortness of breath	What is tuberculosis and how does it affect the lungs	Your experience of living with and after TB	Co-diseases and their impact	Pulmonary rehabilitation: the role of exercise in building strength and endurance	Cigarette smoking and exposure to biomass smoke
Upper body resistance training	Weights, Theraband	Weights, Theraband	Weights, Theraband	Weights, Theraband	Weights, Theraband	Weights, Theraband
Lower body resistance training	Sit-to-stand, stepping up-down	Sit-to-stand, stepping up-down	Sit-to-stand, stepping up-down	Sit-to-stand, stepping up-down	Sit-to-stand, stepping up-down	Sit-to-stand, stepping up-down
Aerobic training	Walking, cycling	Walking, cycling	Walking, cycling	Walking, cycling	Walking, cycling	Walking, cycling

For peer review only

Supplementary Table 2. Physical activity data collection and accelerometry processing criteria

Criteria	Details
Accelerometer Model	ActiGraph wGT3X-BT (version 6.13.4; firmware 1.9.2)
Serial number range	Twenty unique devices will be used; ranging from MOS2E09190617 to MOS2E25190750 and averaging six deployments per device (same serial used for baseline and follow-up wear periods to remove any inter-device variability)
Piezosensor orientation	Triaxial
Mode setup	Mode 29 (x, y, z, steps, lux)
Original sample rate	100 Hz (.gt3x file format)
Deployment method	Baseline:<br>Fitted by research team on Day 0 (Baseline PR Assessment)<br>Fitted by participant on Day 1<br>Follow-up:<br>Fitted by research team Day 0 (11th/12th session)<br>Fitted by participant on Day 1
Location worn	Anterior hip adjacent to the mid-line of the thigh
Requested days of wear	7 days of free-living (10,080 epochs)
Initialization	Not deployed in delay mode in order to standardised capture of Day 0 (00:00) with stop time based on date of first PR class (baseline) and date of follow-up assessment
Wear instructions	Wear continuously except for sleep and water-based activities
Non-wear appropriation	≥60 min of consecutive 0s with allowance for 2 minutes of interruptions
Valid day criteria	≥8 hours of valid waking wear time
Valid file	≥4 valid days for each of the two time points
Missing data	Data modelling or imputation will not be performed
Epoch length	60 seconds
Intensity classification (absolute)	Uniaxial (x-axis) intensity cut-points as follows: Stationary <100 cpm; Light 100-2019 cpm; Moderate 2020-5998cpm; Vigorous ≥5999cpm (Moderate-to-vigorous ≥2020 cpm)
Intensity classification (relative)	Uniaxial (x-axis) cut-points based on Endurance Shuttle Walk Test performance

For peer review only

Table 1. Schedule of events

	**Baseline**	**6 weeks**	**12 weeks**	
	**Face-to-face**	**Face-to-face**	**Face-to-face**	**Telephone***
Socio-demographics	x			
Age of leaving full time education	x			
Smoking and biomass exposure	x			
Respiratory diagnoses	x			
Spirometry	x			
Hospitalisations in the last 12 months	x			
Comorbidities	x			
Treatments	x			
Physical activity monitoring	x	x		
Anthropometrics	x	x	x	
ISWT and ESWT	x	x	x	
5x sit to stand	x	x	x	
MRC score	x	x	x	x
EQ5D score	x	x	x	x
Pain scores for chest pain	x	x	x	x
CCQ	x	x	x	x
CAT	x	x	x	x
HADS	x	x	x	x
WPAI questionnaire	x	x	x	x

Abbreviations: CAT – The COPD Assessment Test, CCQ – Clinical COPD Questionnaire score, ESWT – Endurance Shuttle Walk Test, HADS – Hospital Anxiety and Depression Scale, ISWT – Incremental Shuttle Walk Test, MRC – Medical Research Council, WPAI – Work Productivity and Activity Impairment Questionnaire

For peer review only

SPIRIT

STANDARD PROTOCOL ITEMS: RECOMMENDATIONS FOR INTERVENTIONAL TRIALS

SPIRIT 2013 Checklist: Recommended items to address in a clinical trial protocol and related documents*

Section/item	Item No	Description	PDF Page number
Title	1	Descriptive title identifying the study design, population, interventions, and, if applicable, trial acronym	4
Trial registration	2a	Trial identifier and registry name. If not yet registered, name of intended registry	4
	2b	All items from the World Health Organization Trial Registration Data Set	Throughout
Protocol version	3	Date and version identifier	4
Funding	4	Sources and types of financial, material, and other support	12
Roles and responsibilities	5a	Names, affiliations, and roles of protocol contributors	4, 12
	5b	Name and contact information for the trial sponsor	11
	5c	Role of study sponsor and funders, if any, in study design; collection, management, analysis, and interpretation of data; writing of the report; and the decision to submit the report for publication, including whether they will have ultimate authority over any of these activities	N/A
	5d	Composition, roles, and responsibilities of the coordinating centre, steering committee, endpoint adjudication committee, data management team, and other individuals or groups overseeing the trial, if applicable (see Item 21a for data monitoring committee)	10
Background and rationale	6a	Description of research question and justification for undertaking the trial, including summary of relevant studies (published and unpublished) examining benefits and harms for each intervention	5,6
	6b	Explanation for choice of comparators	6
Objectives	7	Specific objectives or hypotheses	6
Trial design	8	Description of trial design including type of trial (eg, parallel group, crossover, factorial, single group), allocation ratio, and framework (eg, superiority, equivalence, noninferiority, exploratory)	6
Study setting	9	Description of study settings (eg, community clinic, academic hospital) and list of countries where data will be collected. Reference to where list of study sites can be obtained	6

For peer review only

Eligibility criteria	10	Inclusion and exclusion criteria for participants. If applicable, eligibility criteria for study centres and individuals who will perform the interventions (eg, surgeons, psychotherapists)	7
Interventions	11a	Interventions for each group with sufficient detail to allow replication, including how and when they will be administered	7,8,20
	11b	Criteria for discontinuing or modifying allocated interventions for a given trial participant (eg, drug dose change in response to harms, participant request, or improving/worsening disease)	11
	11c	Strategies to improve adherence to intervention protocols, and any procedures for monitoring adherence (eg, drug tablet return, laboratory tests)	9,19
	11d	Relevant concomitant care and interventions that are permitted or prohibited during the trial	8
Outcomes	12	Primary, secondary, and other outcomes, including the specific measurement variable (eg, systolic blood pressure), analysis metric (eg, change from baseline, final value, time to event), method of aggregation (eg, median, proportion), and time point for each outcome. Explanation of the clinical relevance of chosen efficacy and harm outcomes is strongly recommended	9,10
Participant timeline	13	Time schedule of enrolment, interventions (including any run-ins and washouts), assessments, and visits for participants. A schematic diagram is highly recommended (see Figure)	7,15,16
Sample size	14	Estimated number of participants needed to achieve study objectives and how it was determined, including clinical and statistical assumptions supporting any sample size calculations	8
Recruitment	15	Strategies for achieving adequate participant enrolment to reach target sample size	7
Allocation:			
Sequence generation	16a	Method of generating the allocation sequence (eg, computer-generated random numbers), and list of any factors for stratification. To reduce predictability of a random sequence, details of any planned restriction (eg, blocking) should be provided in a separate document that is unavailable to those who enrol participants or assign interventions	8
Allocation concealment mechanism	16b	Mechanism of implementing the allocation sequence (eg, central telephone; sequentially numbered, opaque, sealed envelopes), describing any steps to conceal the sequence until interventions are assigned	8

Implementation	16c	Who will generate the allocation sequence, who will enrol participants, and who will assign participants to interventions	8
Blinding (masking)	17a	Who will be blinded after assignment to interventions (eg, trial participants, care providers, outcome assessors, data analysts), and how	8
	17b	If blinded, circumstances under which unblinding is permissible, and procedure for revealing a participant's allocated intervention during the trial	8
Data collection methods	18a	Plans for assessment and collection of outcome, baseline, and other trial data, including any related processes to promote data quality (eg, duplicate measurements, training of assessors) and a description of study instruments (eg, questionnaires, laboratory tests) along with their reliability and validity, if known. Reference to where data collection forms can be found, if not in the protocol	9,10
	18b	Plans to promote participant retention and complete follow-up, including list of any outcome data to be collected for participants who discontinue or deviate from intervention protocols	9,15,19
Data management	19	Plans for data entry, coding, security, and storage, including any related processes to promote data quality (eg, double data entry; range checks for data values). Reference to where details of data management procedures can be found, if not in the protocol	10
Statistical methods	20a	Statistical methods for analysing primary and secondary outcomes. Reference to where other details of the statistical analysis plan can be found, if not in the protocol	11
	20b	Methods for any additional analyses (eg, subgroup and adjusted analyses)	11
	20c	Definition of analysis population relating to protocol non-adherence (eg, as randomised analysis), and any statistical methods to handle missing data (eg, multiple imputation)	11
Data monitoring	21a	Composition of data monitoring committee (DMC); summary of its role and reporting structure; statement of whether it is independent from the sponsor and competing interests; and reference to where further details about its charter can be found, if not in the protocol. Alternatively, an explanation of why a DMC is not needed	10
	21b	Description of any interim analyses and stopping guidelines, including who will have access to these interim results and make the final decision to terminate the trial	11

For peer review only - http://bmjopen.bmj.com/site/about/guidelines.xhtml

Harms	22	Plans for collecting, assessing, reporting, and managing solicited and spontaneously reported adverse events and other unintended effects of trial interventions or trial conduct	11
Auditing	23	Frequency and procedures for auditing trial conduct, if any, and whether the process will be independent from investigators and the sponsor	11
Research ethics approval	24	Plans for seeking research ethics committee/institutional review board (REC/IRB) approval	4,11
Protocol amendments	25	Plans for communicating important protocol modifications (eg, changes to eligibility criteria, outcomes, analyses) to relevant parties (eg, investigators, REC/IRBs, trial participants, trial registries, journals, regulators)	11
Consent or assent	26a	Who will obtain informed consent or assent from potential trial participants or authorised surrogates, and how (see Item 32)	7
	26b	Additional consent provisions for collection and use of participant data and biological specimens in ancillary studies, if applicable	N/A
Confidentiality	27	How personal information about potential and enrolled participants will be collected, shared, and maintained in order to protect confidentiality before, during, and after the trial	10
Declaration of interests	28	Financial and other competing interests for principal investigators for the overall trial and each study site	12
Access to data	29	Statement of who will have access to the final trial dataset, and disclosure of contractual agreements that limit such access for investigators	12
Ancillary and post-trial care	30	Provisions, if any, for ancillary and post-trial care, and for compensation to those who suffer harm from trial participation	11
Dissemination policy	31a	Plans for investigators and sponsor to communicate trial results to participants, healthcare professionals, the public, and other relevant groups (eg, via publication, reporting in results databases, or other data sharing arrangements), including any publication restrictions	11
	31b	Authorship eligibility guidelines and any intended use of professional writers	12
	31c	Plans, if any, for granting public access to the full protocol, participant-level dataset, and statistical code	12
Informed consent materials	32	Model consent form and other related documentation given to participants and authorised surrogates	17,18
Biological specimens	33	Plans for collection, laboratory evaluation, and storage of biological specimens for genetic or molecular analysis in the current trial and for future use in ancillary studies, if applicable	N/A

For peer review only - http://bmjopen.bmj.com/site/about/guidelines.xhtml

*It is strongly recommended that this checklist be read in conjunction with the SPIRIT 2013 Explanation & Elaboration for important clarification on the items. Amendments to the protocol should be tracked and dated. The SPIRIT checklist is copyrighted by the SPIRIT Group under the Creative Commons "Attribution-NonCommercial-NoDerivs 3.0 Unported" license.

For peer review only

BMJ Open

Culturally adapted Pulmonary Rehabilitation for adults living with post-tuberculosis lung disease in Kyrgyzstan: Protocol for a randomised controlled trial with blinded outcome measures

Journal:	*BMJ Open*
Manuscript ID	bmjopen-2021-048664.R1
Article Type:	Protocol
Date Submitted by the Author:	20-Nov-2021
Complete List of Authors:	Akylbekov, Azamat; National Center of Cardiology and Internal Medicine named after academician M. Mirrakhimov, Pulmonology; Republican Research center of Pulmonology and Rehabilitation<br>Orme, Mark; University of Leicester, Respiratory Sciences<br>Jones, Amy; University of Leicester, Respiratory Sciences<br>Mademilov, Maamed; National Center of Cardiology and Internal Medicine named after academician M. Mirrakhimov, Pulmonology; Republican Research center of Pulmonology and Rehabilitation<br>Muratbekova, Aibermet; National Center of Cardiology and Internal Medicine named after academician M. Mirrakhimov, Pulmonology; Republican Research center of Pulmonology and Rehabilitation<br>Aidaralieva, Shoira; National Center of Cardiology and Internal Medicine named after academician M. Mirrakhimov, Pulmonology; Republican Research center of Pulmonology and Rehabilitation<br>Mirzalieva, Gulzada; National Center of Cardiology and Internal Medicine named after academician M. Mirrakhimov, Pulmonology; Republican Research center of Pulmonology and Rehabilitation<br>Oleinik, Alena; National Center of Cardiology and Internal Medicine named after academician M. Mirrakhimov, Pulmonology; Republican Research center of Pulmonology and Rehabilitation<br>Magdieva, Kamila; National Center of Cardiology and Internal Medicine named after academician M. Mirrakhimov, Pulmonology; Republican Research center of Pulmonology and Rehabilitation<br>Taalaibekova, Aijan; National Center of Cardiology and Internal Medicine named after academician M. Mirrakhimov, Pulmonology; Republican Research center of Pulmonology and Rehabilitation<br>Rysbek kyzy, Aidai; National Center of Cardiology and Internal Medicine named after academician M. Mirrakhimov, Pulmonology; Republican Research center of Pulmonology and Rehabilitation<br>Yusuf, Zainab; Centre for Exercise and Rehabilitation Science, NIHR Leicester Biomedical Research Centre-Respiratory, University Hospitals of Leicester NHS Trust; University of Leicester, Department of Respiratory Sciences<br>Rupert, Jones; University of Plymouth Faculty of Health and Human Sciences<br>Barton, Andy; South West Research Design Service,<br>Miah, Ruhme ; University of Leicester, Department of Respiratory Sciences

	Manise, Adrian ; University of Leicester, Department of Respiratory Sciences<br>Matheson, Jesse; University of Sheffield, Department of Economics<br>Malcolm, Dominic; University of Loughborough<br>Free, Robert; Leicester NIHR Biomedical Research Centre (Respiratory theme), ; University of Leicester, Infection, Inflammation and Immunity<br>Steiner, Michael; Institute for Lung Health, NIHR Leicester Biomedical Research Centre – Respiratory, University of Leicester, Glenfield Hospital,<br>Sooronbaev, Talant; National Center of Cardiology and Internal Medicine named after academician M. Mirrakhimov, Pulmonology; Republican Research center of Pulmonology and Rehabilitation<br>Singh, Sally; University Hospitals of Leicester NHS Trust, Cardiac/Pulmonary Rehabilitation
**Primary Subject Heading**:	Rehabilitation medicine
Secondary Subject Heading:	Respiratory medicine, Infectious diseases, Qualitative research, Sports and exercise medicine
Keywords:	Rehabilitation medicine < INTERNAL MEDICINE, RESPIRATORY MEDICINE (see Thoracic Medicine), Respiratory infections < THORACIC MEDICINE, Tuberculosis < INFECTIOUS DISEASES, Thoracic medicine < INTERNAL MEDICINE, INTERNAL MEDICINE

SCHOLARONE™
Manuscripts

Culturally adapted Pulmonary Rehabilitation for adults living with post-tuberculosis lung disease in Kyrgyzstan: Protocol for a randomised controlled trial with blinded outcome measures

Azamat Akylbekov[1], Mark W. Orme[2,3], Amy V. Jones[2,3], Maamed Mademilov[1], Aibermet Muratbekova[1], Shoira Aidaralieva[1], Gulzada Mirzalieva[1], Alena Oleinik[1], Kamila Magdieva[1], Aijan Taalaibekova[1], Aidai Rysbek kyzy[1], Zainab K. Yusuf[2,3], Rupert Jones[4], Andy Barton[4], Ruhme Miah[2,3], Adrian Manise[5], Jesse Matheson[6], Dominic Malcolm[7], Robert C. Free[2], Michael C. Steiner[2,3], Talant Sooronbaev[1] and Sally J. Singh[2,3]

Affiliations

1. National Institute for Cardiology and Internal Medicine (NCCIM), Bishkek, Kyrgyzstan

2. Department of Respiratory Sciences, University of Leicester, United Kingdom

3. Centre for Exercise and Rehabilitation Science (CERS), NIHR Leicester Biomedical Research Centre – Respiratory, University Hospitals of Leicester NHS Trust – Leicester, United Kingdom

4. Faculty of Health, University of Plymouth, United Kingdom

5. NIHR Leicester Biomedical Research Centre, University Hospitals of Leicester NHS Trust – Leicester, United Kingdom

6. Department of Economics, University of Sheffield, Sheffield, United Kingdom

7. School of Sport, Exercise and Health Sciences, Loughborough University

Protocol Version 1 23/10/2019

Contributorship statement

Azamat Akylbekov is a main author.

Mark W. Orme - Help in all sections of writing the Study Protocol.

Amy V. Jones - Work on the structure of the manuscript and its skeleton

Maamed Mademilov - Work on qualitative part of the Study Protocol

Aibermet Muratbekova - Worked on the data analysis section

Shoira Aidaralieva - Work on REDCap database adaptation

Gulzada Mirzalieva - Assistance in including the qualitative part in the protocol

Alena Oleinik - Help in developing a structure of pulmonary rehabilitation programme

Kamila Magdieva - Help in developing an exercise and educational programmes

Aijan Taalaibekova - Help in developing an educational programme

Aidai Rysbek kyzy - Help in developing an exercise programme

Zainab K. Yusuf - Editorial work and a lot of work on the description of qualitative study

Rupert Jones - The inspirer of new ideas and unconventional approaches

Andy Barton - Editorial work

Ruhme Miah - Technical support and editorial work

Adrian Manise - Database and storage organisation

For peer review only

Jesse Matheson - Technical support and editorial work

Dominic Malcolm - Work on edition of qualitative section

Robert C. Free - Technical support and editorial work

Michael C. Steiner - Author of the main conceptual ideas

Talant Sooronbaev - Author of the idea and mastermind of the research protocol

Sally J. Singh - Co-author of the main idea

ABSTRACT

Introduction: Pulmonary rehabilitation (PR) is a programme of individually prescribed physical exercise, education, and self-management activities. PR is recommended in international guidelines for managing COPD and other chronic respiratory diseases. PR is still under-recognised in tuberculosis (TB) guidelines and PR is not available in many low- and middle income countries (LMIC) and for people with post-TB lung disease (PTBLD). The main aims of the study are to adapt and define a culturally appropriate PR programme in Kyrgyzstan for people living with PTBLD and to test, in a fully powered randomised controlled trial (RCT), the effectiveness of PR in improving exercise capacity for people living with PTBLD.

Methods and analysis: The study will be divided into three stages: *Stage 1:* Focus group discussions with patients living with PTBLD and interviews with PR referrers will be conducted to explore initial perceptions and inform the cultural adaptation, structure and content of PR. *Stage 2a:* A single-blind RCT evaluating the effectiveness of a culturally-adapted 6 week PR programme on maximal exercise capacity, assessed by the incremental shuttle walking test, before and after PR. Participants will be additionally followed-up 12 weeks post-baseline. Additional outcomes will include health-related quality of life, respiratory symptoms, psychological wellbeing and physical function. *Stage 2b:* Participants' experience of PR will be collected through interviews and using a log book and a patient evaluation form. Staff delivering PR will be interviewed to explore their experience of delivering the intervention and refining the delivery for future implementation.

Ethics and dissemination: The study was approved 22/07/2019 by Ethics Committee NCCIM (ref: no. 17) and by University of Leicester ethics committee (ref: no. 22293). Study results will be disseminated through appropriate peer-reviewed journals, national and international respiratory/physiotherapy conferences, social media, and through patient and public involvement events in Kyrgyzstan and in the UK.

Trial registration: International Standard Randomised Controlled Trials Numbers: ISRCTN11122503

Main author's contact email: azamatti@yahoo.com

Strengths and limitations of this study

- This project will inform an appropriate pulmonary rehabilitation programme for people living with post-tuberculosis lung disease (PTBLD) in Kyrgyzstan. This study is the first fully powered randomised controlled trial (RCT) of a culturally appropriate pulmonary rehabilitation programme for adults with PTBLD in Central Asia.
- This is a single-centre fully powered RCT; whilst findings will be important for assessing impact of pulmonary rehabilitation in Kyrgyzstan, Central Asia and low- and middle-income countries (LMIC) more broadly, findings may not be generalizable to other parts of the world.
- Taking a mixed-method approach, this study will provide a rich insight into the experiences of pulmonary rehabilitation for people living with PTBLD.
- Using a common set of outcome measures specifically designed for pulmonary rehabilitation in LMIC will allow for direct comparisons of outcomes between sites across the world.

For peer review only

INTRODUCTION

Background and rationale

Tuberculosis (TB) is an infectious disease and a major cause of death worldwide [1]. It typically affects the lungs (pulmonary TB) and approximately a quarter of people globally are at risk of developing TB [1]. Every year approximately 10 million people worldwide develop TB [2], of which 500,000 are multi-drug resistant cases [3]. Pulmonary TB remains an important cause of chronic respiratory impairment in low- and middle-income countries (LMIC) and globally. Kyrgyzstan is classified as lower-middle income country, is situated in Central Asia and has a higher respiratory mortality compared to Europe, with a TB incidence of 100:100,000. [4]

The presence of long-term respiratory sequelae following Pulmonary TB treatment is well established. [5] [6] The persistence of abnormal airway physiology [7], specifically fixed airways obstruction after treatment, has been documented in large population-based cross-sectional studies. The consequence of suffering from Pulmonary TB is termed post-TB lung disease (PTBLD). PTBLD negatively impacts people's quality of life, with exercise intolerance, respiratory symptoms, and abnormal lung function. [8][9] PTBLD also poses a significant economic burden to individuals and societies, not only attributable to direct costs of drugs and hospital stay, but also indirect costs such as missed workdays or lower productivity. The burden of PTBLD alongside the economic conditions in LMIC such as Kyrgyzstan aggravates the situation of patients, leading to disability and early mortality. [10]

Pulmonary Rehabilitation (PR) is a programme of individually prescribed physical exercise, education, and self-management activities. PR is recommended in international guidelines for managing COPD and other lung diseases [11] but is still under-recognised in TB guidelines and in LMIC. [12] With the established benefits of PR for people living with chronic respiratory diseases many high-income countries have integrated PR services within routine healthcare services. Delivery of PR is encouraged in LMIC where there is profound need [13], where there is emerging evidence that it is feasible to deliver PR in low-resource settings [14] [15], including for people living with PTBLD [23] for whom post-treatment support for health and wellbeing is needed. [16] Indeed, PR is typically delivered by a multidisciplinary team which has been advocated for people living with PTBLD in order to account for psychosocial and economical challenges. [17] [18] Western models of PR may not be optimal in LMIC and there is a need to implement PR interventions tailored to the local culture, traditions, geography, population, and healthcare systems. To date, despite the potential to improve patient outcomes in PTBLD there has been no formal fully-powered trial of PR for people living with PTBLD in LMIC. It is important for PR to be appealing to patients and to ensure it is delivered in a manner sensitive to local contexts. Accordingly, the aim of this study is to adapt a conventional model of PR to Kyrgyzstan and PTBLD contexts and test its effectiveness in a fully powered randomised controlled trial (RCT).

The trial is comprised of: Stage 1: Adaptations to PR informed by patients living with PTBLD and healthcare professionals (HCPs) who would refer to PR; Stage 2a: fully powered RCT; and Stage 2b: qualitative evaluation of PR. The objectives of this study are to:

1. Explore the views of people living with PTBLD and HCPs (referrers and deliverers) involved in the care of patients living with PTBLD to inform the adaptations needed for a PR programme suitable for people living with PTBLD in Kyrgyzstan.
2. Conduct a single-blind fully powered RCT to assess the effectiveness of adapted 6-week PR on maximal exercise capacity (assessed by the incremental shuttle walking test (ISWT)).
3. Evaluate a range of secondary outcome measures including health-related quality of life, respiratory symptoms, functional status and psychological wellbeing following PR.
4. Assess any further changes in all outcome measurements 12 weeks post-baseline.
5. Assess the acceptability of PR of participants and the staff delivering PR to inform further improvements to the service.

For peer review only

METHODS AND ANALYSIS

Trial design

The trial will be conducted, analysed and reported according to the Standard Protocol Items: Recommendations for Interventional Trials (SPIRIT) statement and the trial has been prospectively registered (ISRCTN11122503).

Stage 1

Focus groups with adults living with PTBLD and interviews with staff who would be involved in referring patients to PR will be conducted to explore initial perceptions of PR and to inform the cultural adaptation of PR, for their insight, any minor modifications and any additional needs so that it is suitable for this group and for understand their views on any specific topics for the health education component, or any types of exercises that are suitable for enjoyment and adherence for this population.

Stage 2a

After the PR programme has been adapted based on the qualitative work at stage 1, a RCT evaluating the effectiveness of PR in adults with PTBLD. Adults living with PTBLD will be randomly allocated into one of two groups: Group A (assigned PR programme) or Group B (usual care on PR waiting list).

Stage 2b

Upon completion of PR, patient focus groups and interviews with staff who were involved in the delivery of PR will be conducted. Participants in the PR group will be asked to log their experience as they progress through the programme by completing a log book accessible before, during and after sessions, as well as a dedicated patient evaluation form to be completed after their last class.

Study setting

The study will be conducted at the National Center for Cardiology and Internal Medicine (NCCIM), Bishkek, Kyrgyzstan. Interviews will be conducted in quiet and familiar settings, for example a HCP's office. Spacious and quiet rooms will be used for focus groups. For the PR program, a specially allocated room with the necessary equipment and air conditioner will be used. For all stages of the project, appropriate precautions will be taken in line with local COVID-19 guidance.

Participants

For Stage 1, eligible patients will be: aged ≥18 years; confirmed diagnosis of a TB-negative patients with PTBLD using a Ziehl-Nielsen stain or GeneExpert method and completed TB treatment. Eligible HCPs will be directly involved in the long-term care of PTBLD patients.

For Stage 2a, the inclusion criteria for patients will be: aged ≥18 years; confirmed diagnosis of a TB-negative patients with PTBLD using a Ziehl-Nielsen stain or GeneExpert method; completed TB treatment; Medical Research Council (MRC) dyspnoea score grade 2 or higher. Exclusion criteria for patients will be: Comorbidities such as severe or unstable cardiovascular, other internal diseases and locomotor difficulties that preclude exercise; malignant disease such as lung cancer; evidence of active TB on Chest X-ray or sputum tests within 1 month of assessment; unable or unwilling to provide informed consent.

For Stage 2b, all patients consented and randomised to receive PR and all staff involved in the delivery of PR to patients as part of the trial will be eligible.

Procedure

After receiving a research fact sheet, patients eligible for the study will be asked if they would like to participate after they have had the opportunity to ask questions. If they wish to take part, they will be asked to provide written informed consent (Appendix A). Reasons for declining the study will be taken as field notes. If they wish

For peer review only

to participate, they will be asked to provide written informed consent. Reasons for declining to participate will be reflected in the field notes. The staff of the family medicine centres, doctors of the City Tuberculosis Hospital, the City Tuberculosis Dispensary, the National Hospital, District Hospitals and the National Centre of Cardiology and Internal Medicine will screen patients for eligibility and refer interested patients to the PR study team. Interested patients will be screened by the study team to confirm interest in participation and to schedule the baseline visit. After providing written informed consent, participants will undergo a baseline assessment at the NCCIM by a specially trained team of researchers and residents. After the baseline measures are complete, participants will be randomised into one of two groups: PR or usual care control. At 6-weeks post-baseline and 12-weeks post-baseline, patients will undergo follow-up assessments. If participants are not able to attend follow-up assessments in person, questionnaires only will be completed via telephone appointment. Experiences of the patients living with PTBLD and PR deliverers will be explored in interviews and focus groups. Participants who drop out of the trial will be asked to take part in an interview and information provided freely by the participants will be collected as field notes. All outcome measures will be performed at 6 and 12 weeks by dedicated staff who are blinded to group allocations. Control participants will be offered PR at the end of the study (see Figure 1).

Intervention

The PTBLD PR programme will be held twice-weekly for a period of 6-weeks. Each session will last approximately two hours, consisting of exercise and health education (Supplementary Table 1). The PR programme recruitment will be rolling rather than fixed groups, with a maximum capacity of five patients per session (due to pandemic), and supervised by a nurse, doctor, researcher or physiotherapist. The equipment used will be simple and based on local availability. It will be based on the core elements of an evidence-based rehabilitation, including exercises and health education but we will be adapting the detail and delivery based on the qualitative work in Stage 1.

Core components of PR exercise are upper and lower body resistance training and aerobic training will be informed by international guidelines. [11] [19] The strength exercises will include (at least) bicep curls, pull-ups, sit-to-stands and step ups. Patients will be asked to do one session of strength training and to walk every day at home in addition to structured classes. Minimal equipment will be used where possible, such as bottles filled with water, to achieve the desired weight in kilograms and to offer insight into how participants can exercise effectively at home during and after finishing PR. Aerobic exercise will include walking and static cycling. Walking will be individually prescribed at a speed equivalent to 85% maximal oxygen capacity (VO_2) peak derived from the ISWT. [20] [21] Walking will be monitored and target duration increased by the instructor as the programme progresses. The content delivered within the education sessions will be based on existing materials, with refinement from Stage 1 of this trial and will be delivered by an interdisciplinary team.

Usual care

As part of usual care, participants will receive their usual prescription medications (if appropriate), and an educational booklet regarding PTBLD and the importance of exercise, healthy diet, smoking cessation and avoiding biomass smoke.

Sample size

Stage 1

We will conduct 8-10 focus groups of 2-10 patients (ideally 6-8 patients) in each group and up to 15 interviews with referrers to explore initial perceptions of PR and to inform the cultural adaptation of PR, for their insight, any minor modifications and any additional needs so that it is suitable for this group and for understand their views on any specific topics for the health education component, or any types of exercises that are suitable for enjoyment and adherence for this population.

Stage 2a

For peer review only

Assuming a statistical significance level of 5% and a statistical power of 80%, in order to detect a 35m difference in ISWT measured at baseline and after completion of PR, [22] a total of 114 participants are required (PR: 57 participants or waiting list control: 57 participants). This power calculation was performed using a paired t-test and assumes the ISWT follows an approximately normal distribution. Data from a feasibility study in Uganda, assessing PR in a PTBLD population has guided this calculation. [14] Conservatively assuming up to 30% loss to follow-up at 6-weeks, this will require up to 163 participants to be recruited and randomised (1:1) to each arm. The estimated 70% ineligibility rate suggests approximately 543 PTBLD patients will be screened for this trial.

Stage 2b

We will conduct up to 5 focus groups of 2-10 patients (ideally 6-8 patients) and an interview with all members of the PR delivery team. All participants randomised to the PR group will be asked to complete the log book and evaluation form.

Randomisation

Randomisation will occur through a web based service Sealed Envelope™ and conducted in a designated room by a dedicated member of the research team. Participants will be randomised (1:1) to either PR or usual care. A dedicated member of the research team will inform the assessors of group allocations via telephone. Participants will be informed at the end of their baseline visit to arrange their first PR class.

Blinding

Outcome measures taken at baseline, post-PR, and follow-up will be taken by a researcher blinded to group allocation. It is not possible to blind participants to treatment due to the nature of a PR intervention. Research staff will be blinded to outcome measures. Participants will be advised not to reveal their group during the follow-up assessments. Episodes of un-blinding will be documented and reported for both time points.

Qualitative data collection

All interview and focus group discussions will be audio-recorded, expected to last on average 20-40 minutes, and will be conducted by a trained interviewer. Recordings will be transcribed verbatim, with identifiable information removed. Consent will be obtained from participants prior to their involvement. PR deliverers will be invited to participate in in-depth interviews at the end of the study to discuss aspects of PR, such as insights into barriers to attendance, logistical barriers of running a PR programme and their views of patients' experiences of the intervention.

Book of testimonies and evaluation form

Participants within PR will be asked to log their experience of PR as they progress through the programme. This will be in the form of a PR log book accessible to participants before, during and after sessions, as well as a dedicated evaluation form (Appendix B). Staff involved in PR will also receive the same evaluation form at the end of the study.

Quantitative data collection

Baseline characteristics will comprise patient socio-demographics (age, sex), age of leaving full time education, anthropometrics (height, weight, body mass index), smoking status and pack years, biomass fuel exposure, primary respiratory diagnosis, secondary respiratory diagnosis (if appropriate), spirometry data (post bronchodilation Forced Expiratory Volume in 1-second [FEV_1], post bronchodilation Forced Vital Capacity [FVC] and FEV_1/FVC ratio) (EasyOne™ Plus spirometer, NDD Medical Technologies, Switzerland); number of hospitalisations in the last 12 months, comorbidities, treatment and previous participation in PR. The height will be measured by stadiometer and the weight will be measured by electronic scales.

Outcomes

For peer review only

A schedule of events is provided in Table 1 and measures are in accordance with the recommended minimum dataset for PR in LMIC. [23] In the case a face-to-face follow up appointment at 12 weeks post-baseline cannot be conducted, questionnaires will be collected over telephone.

Primary outcome

The primary outcome of exercise capacity will be measured using the ISWT. [20] The ISWT will require patients to walk up and down a 10-meter course marked out using cones at walking speeds dictated by an audio signal played on an audio device. Each participant will receive standardised instructions to: "Walk at a steady pace, aiming to turn around when you hear the signal. You should continue to walk until you feel that you are unable to maintain the required speed without becoming unduly breathless". To account for any learning effect, a practice ISWT will be performed and the greatest distance walked from either test will be taken forward. The test will be terminated when either: (i) the patient indicates that they are unable to continue, (ii) the operator determines that the patient is not fit to continue or (iii) the operator assesses that the patient was unable to sustain the speed and cover the distance to the cone prior to the beep sounding.

Secondary outcomes

Exercise capacity and muscular strength

Participants will be asked to complete a five repetition sit to stand test, [24] as an indication of lower extremity strength. The time taken for participants to complete this task will be recorded.

The endurance shuttle walk test (ESWT) is a constant load walking test, in which a participant is required to walk at 85% of their maximal ISWT walking speed. An audio signal is played to the participant and they are required to walk around a 10-meter course marked by cones. [21]

Symptoms

The MRC Dyspnoea Scale will be used to assess dyspnoea. [25] This is a 5-point questionnaire, in which the participant self-reports their dyspnoea. Chest pain will be assessed using four questions based on Brief Pain Inventory (Short Form). [26]

Quality of life

The Clinical COPD Questionnaire (CCQ) [27] is a 10-item health related quality of life questionnaire that is divided into three domains: symptoms, functional and mental. The COPD Assessment Test (CAT) [28] is a self-administered questionnaire that is used to quantify the symptom burden of COPD. The questionnaire consists of eight items, each with a 6-point scale, creating a total score out of 40. The EQ5D5L [29] will be used to assess generic health-related quality of life, and comprises of five dimensions: mobility, self-care, usual activities, pain/discomfort and anxiety/depression. The questionnaire also contains a visual analogue scale, where the participant identifies their health from 'the best you can imagine' and the 'worst you can imagine'.

Psychological wellbeing

The self-reported Hospital Anxiety and Depression Scale [30] will be used to determine levels of anxiety and depression. The questionnaire consists of 14 questions with a 4-point Likert scale, and two 7-item subscales for anxiety and depression.

Productivity

The Work Productivity and Activity Impairment (WPAI) questionnaire [31] will be used to assess productivity and impairments in paid/ unpaid work and activities. The questionnaire is self-reported and participants are asked to recall the time missed from work in the last seven days.

For peer review only

Physical activity

Physical activity will be assessed using an ActiGraph wGT3X-BT activity monitor (ActiGraph, Pensacola, FL, USA). Participants will be asked to place the device on their right anterior hip during waking hours for one week prior to attending PR (baseline) and for one week prior to their 6-week follow-up visit. Participants will be asked to remove the device for water-based activities and for sleep. Written instructions will be provided to the participants. Full methodology for the assessment of physical activity is provided in Supplementary Table 2.

Cost/benefit analysis

The cost of the PR programme will be calculated, including single and recurrent costs. Single costs will include the necessary costs to set up and run PR and the recurrent costs will include any item with a life expectancy of no more than 1 year e.g. disposable materials. Fixed costs will be captured before the first patient being recruited into the trial. Recurrent costs will be collected at the halfway stage of recruitment. An average cost of PR per patient will be calculated.

Data management

Data collected during the study will be entered onto a database using Research Electronic Data Capture (REDCap), [32] [33] hosted by University of Leicester, UK. Access to the database will be via a secure password protected web-interface. Data will be validated using real-time data entry validation and electronic checks, led by the Independent Data Monitoring Committee (IDMC) at the University of Leicester, UK. The participants will be allocated a unique study-specific identification code.

Data analysis

For Stages 1 and 2b, qualitative data will be transcribed verbatim in Russian and translated into English for triangulation with UK-based researchers. Transcripts will be analysed using Thematic Analysis; following the six distinct stages of familiarization with data; generating initial codes; searching for themes; reviewing themes; defining and naming themes and producing the report. [34] [35] The qualitative lead will carry out initial coding and a sample of the transcripts will be coded independently by a second researcher to improve consistency and interpretive authenticity. The team will meet regularly to review emerging themes, with close attention to interactions within the interview and focus group data.

For Stage 2a, quantitative analysis will be conducted using Statistical Package for the Social Sciences. Data will be reported as mean (standard deviation (SD)), median (interquartile range (IQR)) or frequency (%) as appropriate. All randomised patients will be included in an intention to treat analysis with the primary efficacy analysis based on both per protocol and modified intention-to-treat populations, with missing data imputed. For the primary analysis, the differences in the primary outcome of walking distance on the ISWT will be estimated using mixed models (unadjusted and adjusted for covariates if there are differences between groups at baseline). There will be no formal interim analysis of the data.

Adverse events

All adverse events and serious adverse events will be recorded on an adverse event log. This will be recorded within the study trial management paperwork, CRF and study database. The IDMC will review high level safety data which will be monitored at least every month and on an ad hoc basis as required. The NIHR RECHARGE Scientific Committee will be informed of all adverse events and determine the need to terminate the trial prematurely. Participants who experience any such event will receive the appropriate care.

Patient and public involvement

For peer review only

We know from conversations with adults living with PTBLD that support after completing TB treatment is lacking and that these individuals continue to live with a reduce quality of life, including respiratory symptoms and social isolation. It is clear that these individuals would benefit from support to better manage their condition, which is what this project seeks to deliver. The PR intervention and trial are to be informed directly from people living with PTBLD and healthcare workers in Kyrgyzstan. The results of this work will be presented to patients and the public at dedicated dissemination events, including those hosted at the NCCIM, Bishkek and surrounding TB Hospitals.

ETHICS AND DISSEMINATION

Research ethics approval

The study was approved 22/07/2019 by Ethics Committee of the NCCIM (ref: no. 17) 3, Togolok Moldo Street, Bishkek, Kyrgyzstan. Ethics approval was also provided by the University of Leicester ethics committee on the 16/09/2019 ((reference number 22293, ethicsapp@leicester.ac.uk). Information about the study participants will be strictly confidential, and the names will be used only for internal reporting. No identifiable data will be published in any article.

Dissemination policy

Study results will be disseminated through appropriate peer-reviewed journals, national and international conferences, and through social media. All participants will be provided a summary of the trial results. In February 2021, a National Scientific Medical Forum is planned and a National Respiratory Congress is planned in October 2021, which will also present the results of the study and disseminate information about the project. In addition, mobile rehabilitation schools are planned during 2021.

AUTHORS' CONTRIBUTIONS

All authors (Azamat Akylbekov, Mark W. Orme, Amy V. Jones, Maamed Mademilov, Aibermet Muratbekova, Shoira Aidaralieva, Gulzada Mirzalieva, Alena Oleinik, Kamila Magdieva, Aijan Taalaibekova, Aidai Rysbek kyzy, Zainab K. Yusuf, Rupert Jones, Andy Barton, Ruhme Miah, Adrian Manise, Jesse Matheson, Robert C. Free, Michael C. Steiner, Talant Sooronbaev, Sally J. Singh) have substantially contributed to the conception and design of the study. Azamat Akylbekov, Mark W. Orme and Amy V. Jones drafted the manuscript. All authors of the paper have revised the content and approved the final version to be published. All authors are accountable for all aspects of the work.

ROLES AND RESPONSIBILITIES OF AUTHORS

a) Substantially contributed to the conception and/ or design of the work

b) Substantially contributed to the acquisition, analysis, or interpretation of data for the work

c) Substantially contributed to the drafting of the work and/ or revising it critically for important intellectual content

FUNDING STATEMENT

This research was funded by the National Institute for Health Research (NIHR) (17/63/20) using UK aid from the UK Government to support global health research. The views expressed in this publication are those of the author(s) and not necessarily those of the NIHR or the UK Department of Health and Social Care.

COMPETING INTERESTS

None declared

DATA ACCESS

For peer review only

Following the completion of the project, data from the Global Health Research Group on Respiratory Rehabilitation (Global RECHARGE) Core Dataset will be made available to the wider community upon reasonable request.

REFERENCES

[1] "World Health Organisation. Global tuberculosis report. 2020.," 2020. https://www.who.int/tb/publications/global_report/en/.

[2] United Nations General Assembly. Resolution 73/3, "Political declaration of the high-level meeting of the General Assembly on the fight against tuberculosis. United Nations," *Int. Organ.*, vol. 9, no. 1, pp. 57–156, 2018, doi: 10.1017/s002081830002244x.

[3] M. Uplekar *et al.*, "Viewpoint WHO ' s new End TB Strategy," pp. 1799–1801, 2015, doi: 10.1016/S0140-6736(15)60570-0.

[4] WHO, "Tuberculosis profile: Kyrgyzstan," 2019. https://worldhealthorg.shinyapps.io/tb_profiles/?_inputs_&entity_type=%22country%22&lan=%22EN%22&iso2=%22KG%22.

[5] A. L. Byrne, B. J. Marais, C. D. Mitnick, L. Lecca, and G. B. Marks, "Tuberculosis and chronic respiratory disease: A systematic review," *Int. J. Infect. Dis.*, vol. 32, pp. 138–146, 2015, doi: 10.1016/j.ijid.2014.12.016.

[6] B. W. Allwood, L. Myer, and E. D. Bateman, "A systematic review of the association between pulmonary tuberculosis and the development of chronic airflow obstruction in adults," *Respiration*, vol. 86, no. 1, pp. 76–85, 2013, doi: 10.1159/000350917.

[7] K. Dheda, H. Booth, J. F. Huggett, M. A. Johnson, A. Zumla, and G. A. W. Rook, "Lung remodeling in pulmonary tuberculosis," *J. Infect. Dis.*, vol. 192, no. 7, pp. 1201–1210, 2005, doi: 10.1086/444545.

[8] S. Daniels, Kurt J.; Irusen, Elvis; Pharaoh, Hamilton; Hanekom, "Post-tuberculosis health-related quality of life, lung function and exercise capacity in a cured pulmonary tuberculosis population in the Breede Valley District, South Africa," *South African J. Physiother.*, no. English 2009, pp. 1–8, 2013.

[9] J. Meghji *et al.*, "Patient outcomes associated with post-tuberculosis lung damage in Malawi: A prospective cohort study," *Thorax*, vol. 75, no. 3, pp. 269–278, 2020, doi: 10.1136/thoraxjnl-2019-213808.

[10] K. Romanowski, B. Baumann, C. A. Basham, F. Ahmad Khan, G. J. Fox, and J. C. Johnston, "Long-term all-cause mortality in people treated for tuberculosis: a systematic review and meta-analysis," *Lancet Infect. Dis.*, vol. 19, no. 10, pp. 1129–1137, 2019, doi: 10.1016/S1473-3099(19)30309-3.

[11] M. A. Spruit *et al.*, "An official American thoracic society/European respiratory society statement: Key concepts and advances in pulmonary rehabilitation," *Am. J. Respir. Crit. Care Med.*, vol. 188, no. 8, 2013, doi: 10.1164/rccm.201309-1634ST.

[12] A. Wanner *et al.*, "International research and guidelines on post-tuberculosis chronic lung disorders: A systematic scoping review," *BMJ Glob. Heal.*, vol. 3, no. 4, pp. 1–8, 2018, doi: 10.1136/bmjgh-2018-000745.

[13] S. J. Singh, D. M. G. Halpin, S. Salvi, B. J. Kirenga, and K. Mortimer, "Exercise and pulmonary rehabilitation for people with chronic lung disease in LMICs: challenges and opportunities.," *Lancet. Respir. Med.*, vol. 7, no. 12, pp. 1002–1004, Dec. 2019, doi: 10.1016/S2213-2600(19)30364-9.

[14] R. Jones *et al.*, "A pre – post intervention study of pulmonary rehabilitation for adults with post-tuberculosis lung disease in Uganda," pp. 3533–3539, 2017.

[15] M. Anastasaki *et al.*, "Establishing a pulmonary rehabilitation programme in primary care in Greece: A FRESH AIR implementation study," *Chron. Respir. Dis.*, vol. 16, 2019, doi: 10.1177/1479973119882939.

For peer review only

[16] B. Allwood *et al.*, “The long shadow post-tuberculosis,” *Lancet Infect. Dis.*, vol. 19, no. 11, pp. 1170–1171, 2019, doi: https://doi.org/10.1016/S1473-3099(19)30564-X.

[17] S. Datta and C. A. Evans, “Healthy survival after tuberculosis,” *Lancet Infect. Dis.*, vol. 19, no. 10, pp. 1045–1047, 2019, doi: https://doi.org/10.1016/S1473-3099(19)30387-1.

[18] T. Wingfield, M. A. Tovar, S. Datta, M. J. Saunders, and C. A. Evans, “Addressing social determinants to end tuberculosis,” *Lancet*, vol. 391, no. 10126, pp. 1129–1132, 2018, doi: 10.1016/S0140-6736(18)30484-7.

[19] C. E. Bolton *et al.*, “British Thoracic Society guideline on pulmonary rehabilitation in adults.,” *Thorax*, vol. 68 Suppl 2, pp. ii1-30, Sep. 2013, doi: 10.1136/thoraxjnl-2013-203808.

[20] S. J. Singh, M. D. Morgan, S. Scott, D. Walters, and A. E. Hardman, “Development of a shuttle walking test of disability in patients with chronic airways obstruction.,” *Thorax*, vol. 47, no. 12, pp. 1019–1024, Dec. 1992, doi: 10.1136/thx.47.12.1019.

[21] S. M. Revill, M. D. Morgan, S. J. Singh, J. Williams, and A. E. Hardman, “The endurance shuttle walk: a new field test for the assessment of endurance capacity in chronic obstructive pulmonary disease.,” *Thorax*, vol. 54, no. 3, pp. 213–222, Mar. 1999, doi: 10.1136/thx.54.3.213.

[22] R. A. Evans and S. J. Singh, “Minimum important difference of the incremental shuttle walk test distance in patients with COPD.,” *Thorax*, vol. 74, no. 10, pp. 994–995, Oct. 2019, doi: 10.1136/thoraxjnl-2018-212725.

[23] M. W. Orme *et al.*, “Global RECHARGE: Establishing a standard international data set for pulmonary rehabilitation in low- and middle-income countries.,” *J. Glob. Health*, vol. 10, no. 2, p. 20316, Dec. 2020, doi: 10.7189/jogh.10.020316.

[24] R. W. Bohannon, “Sit-to-stand test for measuring performance of lower extremity muscles.,” *Percept. Mot. Skills*, vol. 80, no. 1, pp. 163–166, Feb. 1995, doi: 10.2466/pms.1995.80.1.163.

[25] J. C. Bestall, E. A. Paul, R. Garrod, R. Garnham, P. W. Jones, and J. A. Wedzicha, “Usefulness of the Medical Research Council (MRC) dyspnoea scale as a measure of disability in patients with chronic obstructive pulmonary disease,” *Thorax*, vol. 54, no. 7, pp. 581–586, 1999, doi: 10.1136/thx.54.7.581.

[26] T. Mendoza, T. Mayne, D. Rublee, and C. Cleeland, “Reliability and validity of a modified Brief Pain Inventory short form in patients with osteoarthritis.,” *Eur. J. Pain*, vol. 10, no. 4, pp. 353–361, May 2006, doi: 10.1016/j.ejpain.2005.06.002.

[27] T. van der Molen, B. W. M. Willemse, S. Schokker, N. H. T. ten Hacken, D. S. Postma, and E. F. Juniper, “Development, validity and responsiveness of the Clinical COPD Questionnaire.,” *Health Qual. Life Outcomes*, vol. 1, p. 13, Apr. 2003, doi: 10.1186/1477-7525-1-13.

[28] P. W. Jones, G. Harding, P. Berry, I. Wiklund, W.-H. Chen, and N. Kline Leidy, “Development and first validation of the COPD Assessment Test,” *Eur. Respir. J.*, vol. 34, no. 3, pp. 648–654, 2009, doi: 10.1183/09031936.00102509.

[29] T. E. Group, “EuroQol--a new facility for the measurement of health-related quality of life.,” *Health Policy*, vol. 16, no. 3, pp. 199–208, Dec. 1990, doi: 10.1016/0168-8510(90)90421-9.

[30] A. S. Zigmond and R. P. Snaith, “The hospital anxiety and depression scale.,” *Acta Psychiatr. Scand.*, vol. 67, no. 6, pp. 361–370, Jun. 1983, doi: 10.1111/j.1600-0447.1983.tb09716.x.

[31] M. C. Reilly, A. S. Zbrozek, and E. M. Dukes, “The validity and reproducibility of a work productivity and activity impairment instrument.,” *Pharmacoeconomics*, vol. 4, no. 5, pp. 353–365, Nov. 1993, doi: 10.2165/00019053-199304050-00006.

[32] P. A. Harris, R. Taylor, R. Thielke, J. Payne, N. Gonzalez, and J. G. Conde, “Research electronic data capture (REDCap)--a metadata-driven methodology and workflow process for providing translational research informatics support.,” *J. Biomed. Inform.*, vol. 42, no. 2, pp. 377–381, Apr. 2009, doi:

For peer review only

10.1016/j.jbi.2008.08.010.

[33] P. A. Harris *et al.*, “The REDCap consortium: Building an international community of software platform partners.,” *J. Biomed. Inform.*, vol. 95, p. 103208, Jul. 2019, doi: 10.1016/j.jbi.2019.103208.

[34] V. Braun and V. Clarke, “Using thematic analysis in psychology,” *Qual. Res. Psychol.*, vol. 3, no. 2, pp. 77–101, 2006, doi: 10.1191/1478088706qp063oa.

[35] D. B. Maguire M, “Doing a Thematic Analysis : A Practical, Step-by-Step Guide for Learning and Teaching Scholars,” *AISHE-J.*, 2017.

For peer review only

Tables

Table 1. Schedule of events

	Baseline	6 weeks	12 weeks	
	**Face-to-face**	**Face-to-face**	**Face-to-face**	**Telephone***
Socio-demographics	x			
Age of leaving full time education	x			
Smoking and biomass exposure	x			
Respiratory diagnoses	x			

Spirometry	x			
Hospitalisations in the last 12 months	x			
Comorbidities	x			
Treatments	x			
Physical activity monitoring	x	x		
Anthropometrics	x	x	x	
ISWT and ESWT	x	x	x	
5x sit to stand	x	x	x	
MRC score	x	x	x	x
EQ5D score	x	x	x	x
Pain scores for chest pain	x	x	x	x
CCQ	x	x	x	x
CAT	x	x	x	x
HADS	x	x	x	x
WPAI questionnaire	x	x	x	x

Abbreviations: CAT – The COPD Assessment Test, CCQ – Clinical COPD Questionnaire score, ESWT – Endurance Shuttle Walk Test, HADS – Hospital Anxiety and Depression Scale, ISWT – Incremental Shuttle Walk Test, MRC – Medical Research Council, WPAI – Work Productivity and Activity Impairment Questionnaire

Figure 1 legend

Research flow. The stages and flow of research are clearly described here.

For peer review only

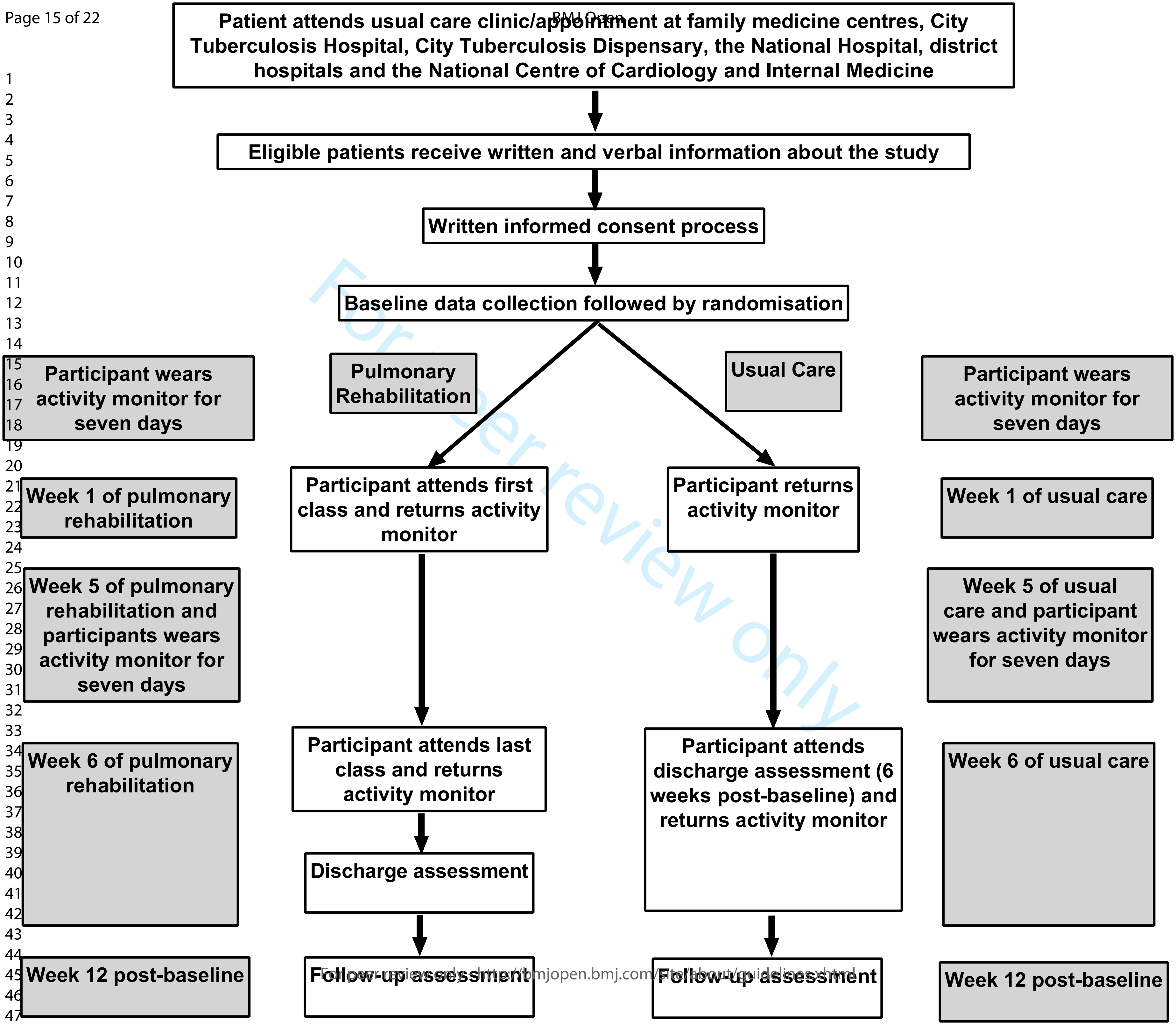

For peer review only

APPENDIX A: Pulmonary Rehabilitation Satisfaction Survey

Please tick the relevant column for your answer to each statement below:	**Strongly Disagree**	**Disagree**	**Neutral**	**Agree**	**Strongly Agree**
I can do more of my daily activities since completing pulmonary rehabilitation.					
My levels of fitness have improved since beginning pulmonary rehabilitation.					
I have found pulmonary rehabilitation to be worthwhile.					
The information in the education talks was useful.					
Pulmonary rehabilitation has helped me to manage my lung condition more effectively.					
I would recommend this pulmonary rehabilitation course to others with a lung condition.					

- **What were the most useful aspects of the course?**

- **Is there anything you feel we could add to the course?**

- **Do you have a comment that that we could use for promotion of the programme which would encourage other patients to participate?**

Thank you very much for taking the time to complete this survey.

For peer review only

Supplementary Table 1. Schedule of PR sessions

Components & duration	Week 1		Week 2		Week 3	
	Class 1	Class 2	Class 3	Class 4	Class 5	Class 6
Education and tea (35 min):<br>• Lectures (15 min)<br>• Questions and feedback (10 min)<br>• Tea (10 min)	Drug treatment and the use of inhalers, as well as why they are prescribed	Healthy nutrition and its role in human life	Strategies for coping with PTBLD	Chest infections and what to do if symptoms worsen	Pulmonary rehabilitation: continuation of the programme at home	Question and Answer session
Upper body resistance training	Weights, Theraband	Weights, Theraband	Weights, Theraband	Weights, Theraband	Weights, Theraband	Weights, Theraband
Lower body resistance training	Sit-to-stand, stepping up-down	Sit-to-stand, stepping up-down	Sit-to-stand, stepping up-down	Sit-to-stand, stepping up-down	Sit-to-stand, stepping up-down	Sit-to-stand, stepping up-down
Aerobic training	Walking, cycling	Walking, cycling	Walking, cycling	Walking, cycling	Walking, cycling	Walking, cycling
**Components & duration**	**Week 4**		**Week 5**		**Week 6**	
	**Class 7**	**Class 8**	**Class 9**	**Class 10**	**Class 11**	**Class 12**
Education and tea (35 min):<br>• Lectures (15 min)<br>• Questions and feedback (10 min)<br>Tea (10 min)	Information about the respiratory system and possible causes of shortness of breath	What is tuberculosis and how does it affect the lungs	Your experience of living with and after TB	Co-diseases and their impact	Pulmonary rehabilitation: the role of exercise in building strength and endurance	Cigarette smoking and exposure to biomass smoke
Upper body resistance training	Weights, Theraband	Weights, Theraband	Weights, Theraband	Weights, Theraband	Weights, Theraband	Weights, Theraband
Lower body resistance training	Sit-to-stand, stepping up-down	Sit-to-stand, stepping up-down	Sit-to-stand, stepping up-down	Sit-to-stand, stepping up-down	Sit-to-stand, stepping up-down	Sit-to-stand, stepping up-down
Aerobic training	Walking, cycling	Walking, cycling	Walking, cycling	Walking, cycling	Walking, cycling	Walking, cycling

For peer review only

Supplementary Table 2. Physical activity data collection and accelerometry processing criteria

**Criteria**	**Details**
Accelerometer Model	ActiGraph wGT3X-BT (version 6.13.4; firmware 1.9.2)
Serial number range	Twenty unique devices will be used; ranging from MOS2E09190617 to MOS2E25190750 and averaging six deployments per device (same serial used for baseline and follow-up wear periods to remove any inter-device variability)
Piezosensor orientation	Triaxial
Mode setup	Mode 29 (x, y, z, steps, lux)
Original sample rate	100 Hz (.gt3x file format)
Deployment method	Baseline:<br>Fitted by research team on Day 0 (Baseline PR Assessment)<br>Fitted by participant on Day 1<br>Follow-up:<br>Fitted by research team Day 0 (11th/12th session)<br>Fitted by participant on Day 1
Location worn	Anterior hip adjacent to the mid-line of the thigh
Requested days of wear	7 days of free-living (10,080 epochs)
Initialization	Not deployed in delay mode in order to standardised capture of Day 0 (00:00) with stop time based on date of first PR class (baseline) and date of follow-up assessment
Wear instructions	Wear continuously except for sleep and water-based activities
Non-wear appropriation	≥60 min of consecutive 0s with allowance for 2 minutes of interruptions
Valid day criteria	≥8 hours of valid waking wear time
Valid file	≥4 valid days for each of the two time points
Missing data	Data modelling or imputation will not be performed
Epoch length	60 seconds
Intensity classification (absolute)	Uniaxial (x-axis) intensity cut-points as follows: Stationary <100 cpm; Light 100-2019 cpm; Moderate 2020-5998cpm; Vigorous ≥5999cpm (Moderate-to-vigorous ≥2020 cpm)
Intensity classification (relative)	Uniaxial (x-axis) cut-points based on Endurance Shuttle Walk Test performance

For peer review only

SPIRIT

STANDARD PROTOCOL ITEMS: RECOMMENDATIONS FOR INTERVENTIONAL TRIALS

SPIRIT 2013 Checklist: Recommended items to address in a clinical trial protocol and related documents*

Section/item	Item No	Description	PDF Page number
Title	1	Descriptive title identifying the study design, population, interventions, and, if applicable, trial acronym	4
Trial registration	2a	Trial identifier and registry name. If not yet registered, name of intended registry	4
	2b	All items from the World Health Organization Trial Registration Data Set	Throughout
Protocol version	3	Date and version identifier	4
Funding	4	Sources and types of financial, material, and other support	12
Roles and responsibilities	5a	Names, affiliations, and roles of protocol contributors	4, 12
	5b	Name and contact information for the trial sponsor	11
	5c	Role of study sponsor and funders, if any, in study design; collection, management, analysis, and interpretation of data; writing of the report; and the decision to submit the report for publication, including whether they will have ultimate authority over any of these activities	N/A
	5d	Composition, roles, and responsibilities of the coordinating centre, steering committee, endpoint adjudication committee, data management team, and other individuals or groups overseeing the trial, if applicable (see Item 21a for data monitoring committee)	10
Background and rationale	6a	Description of research question and justification for undertaking the trial, including summary of relevant studies (published and unpublished) examining benefits and harms for each intervention	5,6
	6b	Explanation for choice of comparators	6
Objectives	7	Specific objectives or hypotheses	6
Trial design	8	Description of trial design including type of trial (eg, parallel group, crossover, factorial, single group), allocation ratio, and framework (eg, superiority, equivalence, noninferiority, exploratory)	6
Study setting	9	Description of study settings (eg, community clinic, academic hospital) and list of countries where data will be collected. Reference to where list of study sites can be obtained	6

For peer review only

Eligibility criteria	10	Inclusion and exclusion criteria for participants. If applicable, eligibility criteria for study centres and individuals who will perform the interventions (eg, surgeons, psychotherapists)	7
Interventions	11a	Interventions for each group with sufficient detail to allow replication, including how and when they will be administered	7,8,20
	11b	Criteria for discontinuing or modifying allocated interventions for a given trial participant (eg, drug dose change in response to harms, participant request, or improving/worsening disease)	11
	11c	Strategies to improve adherence to intervention protocols, and any procedures for monitoring adherence (eg, drug tablet return, laboratory tests)	9,19
	11d	Relevant concomitant care and interventions that are permitted or prohibited during the trial	8
Outcomes	12	Primary, secondary, and other outcomes, including the specific measurement variable (eg, systolic blood pressure), analysis metric (eg, change from baseline, final value, time to event), method of aggregation (eg, median, proportion), and time point for each outcome. Explanation of the clinical relevance of chosen efficacy and harm outcomes is strongly recommended	9,10
Participant timeline	13	Time schedule of enrolment, interventions (including any run-ins and washouts), assessments, and visits for participants. A schematic diagram is highly recommended (see Figure)	7,15,16
Sample size	14	Estimated number of participants needed to achieve study objectives and how it was determined, including clinical and statistical assumptions supporting any sample size calculations	8
Recruitment	15	Strategies for achieving adequate participant enrolment to reach target sample size	7
Allocation:			
Sequence generation	16a	Method of generating the allocation sequence (eg, computer-generated random numbers), and list of any factors for stratification. To reduce predictability of a random sequence, details of any planned restriction (eg, blocking) should be provided in a separate document that is unavailable to those who enrol participants or assign interventions	8
Allocation concealment mechanism	16b	Mechanism of implementing the allocation sequence (eg, central telephone; sequentially numbered, opaque, sealed envelopes), describing any steps to conceal the sequence until interventions are assigned	8

For peer review only - http://bmjopen.bmj.com/site/about/guidelines.xhtml

Implementation	16c	Who will generate the allocation sequence, who will enrol participants, and who will assign participants to interventions	8
Blinding (masking)	17a	Who will be blinded after assignment to interventions (eg, trial participants, care providers, outcome assessors, data analysts), and how	8
	17b	If blinded, circumstances under which unblinding is permissible, and procedure for revealing a participant's allocated intervention during the trial	8
Data collection methods	18a	Plans for assessment and collection of outcome, baseline, and other trial data, including any related processes to promote data quality (eg, duplicate measurements, training of assessors) and a description of study instruments (eg, questionnaires, laboratory tests) along with their reliability and validity, if known. Reference to where data collection forms can be found, if not in the protocol	9,10
	18b	Plans to promote participant retention and complete follow-up, including list of any outcome data to be collected for participants who discontinue or deviate from intervention protocols	9,15,19
Data management	19	Plans for data entry, coding, security, and storage, including any related processes to promote data quality (eg, double data entry; range checks for data values). Reference to where details of data management procedures can be found, if not in the protocol	10
Statistical methods	20a	Statistical methods for analysing primary and secondary outcomes. Reference to where other details of the statistical analysis plan can be found, if not in the protocol	11
	20b	Methods for any additional analyses (eg, subgroup and adjusted analyses)	11
	20c	Definition of analysis population relating to protocol non-adherence (eg, as randomised analysis), and any statistical methods to handle missing data (eg, multiple imputation)	11
Data monitoring	21a	Composition of data monitoring committee (DMC); summary of its role and reporting structure; statement of whether it is independent from the sponsor and competing interests; and reference to where further details about its charter can be found, if not in the protocol. Alternatively, an explanation of why a DMC is not needed	10
	21b	Description of any interim analyses and stopping guidelines, including who will have access to these interim results and make the final decision to terminate the trial	11

Harms	22	Plans for collecting, assessing, reporting, and managing solicited and spontaneously reported adverse events and other unintended effects of trial interventions or trial conduct	11
Auditing	23	Frequency and procedures for auditing trial conduct, if any, and whether the process will be independent from investigators and the sponsor	11
Research ethics approval	24	Plans for seeking research ethics committee/institutional review board (REC/IRB) approval	4,11
Protocol amendments	25	Plans for communicating important protocol modifications (eg, changes to eligibility criteria, outcomes, analyses) to relevant parties (eg, investigators, REC/IRBs, trial participants, trial registries, journals, regulators)	11
Consent or assent	26a	Who will obtain informed consent or assent from potential trial participants or authorised surrogates, and how (see Item 32)	7
	26b	Additional consent provisions for collection and use of participant data and biological specimens in ancillary studies, if applicable	N/A
Confidentiality	27	How personal information about potential and enrolled participants will be collected, shared, and maintained in order to protect confidentiality before, during, and after the trial	10
Declaration of interests	28	Financial and other competing interests for principal investigators for the overall trial and each study site	12
Access to data	29	Statement of who will have access to the final trial dataset, and disclosure of contractual agreements that limit such access for investigators	12
Ancillary and post-trial care	30	Provisions, if any, for ancillary and post-trial care, and for compensation to those who suffer harm from trial participation	11
Dissemination policy	31a	Plans for investigators and sponsor to communicate trial results to participants, healthcare professionals, the public, and other relevant groups (eg, via publication, reporting in results databases, or other data sharing arrangements), including any publication restrictions	11
	31b	Authorship eligibility guidelines and any intended use of professional writers	12
	31c	Plans, if any, for granting public access to the full protocol, participant-level dataset, and statistical code	12
Informed consent materials	32	Model consent form and other related documentation given to participants and authorised surrogates	17,18
Biological specimens	33	Plans for collection, laboratory evaluation, and storage of biological specimens for genetic or molecular analysis in the current trial and for future use in ancillary studies, if applicable	N/A

For peer review only - http://bmjopen.bmj.com/site/about/guidelines.xhtml

*It is strongly recommended that this checklist be read in conjunction with the SPIRIT 2013 Explanation & Elaboration for important clarification on the items. Amendments to the protocol should be tracked and dated. The SPIRIT checklist is copyrighted by the SPIRIT Group under the Creative Commons "Attribution-NonCommercial-NoDerivs 3.0 Unported" license.

For peer review only
